# PARP-14 Promotes Survival of Mammalian α but Not β Pancreatic Cells Following Cytokine Treatment

**DOI:** 10.3389/fendo.2019.00271

**Published:** 2019-05-03

**Authors:** Floriana D'Angeli, Marina Scalia, Matilde Cirnigliaro, Cristina Satriano, Vincenza Barresi, Nicolò Musso, Angela Trovato-Salinaro, Davide Barbagallo, Marco Ragusa, Cinzia Di Pietro, Michele Purrello, Vittoria Spina-Purrello

**Affiliations:** ^1^Department of Biomedical and Biotechnological Sciences, Section of Medical Biochemistry, University of Catania, Catania, Italy; ^2^Department of Biomedical and Biotechnological Sciences, Section of Biology and Genetics, University of Catania, Catania, Italy; ^3^Department of Chemical Sciences, University of Catania, Catania, Italy

**Keywords:** PARP-14, JNK1, JNK2, cytokines, PJ-34, survival

## Abstract

PARP-14 (poly-ADP Ribose Polymerase-14), a member of the PARP family, belongs to the group of Bal proteins (B Aggressive Lymphoma). PARP-14 has recently appeared to be involved in the transduction pathway mediated by JNKs (c Jun N terminal Kinases), among which JNK2 promotes cancer cell survival. Several pharmacological PARP inhibitors are currently used as antitumor agents, even though they have also proved to be effective in many inflammatory diseases. Cytokine release from immune system cells characterizes many autoimmune inflammatory disorders, including type I diabetes, in which the inflammatory state causes β cell loss. Nevertheless, growing evidence supports a concomitant implication of glucagon secreting α cells in type I diabetes progression. Here, we provide evidence on the activation of a survival pathway, mediated by PARP-14, in pancreatic α cells, following treatment of αTC1.6 glucagonoma and βTC1 insulinoma cell lines with a cytokine cocktail: interleukin 1 beta (IL-1β), interferon gamma (IFN-γ) and tumor necrosis factor alpha (TNF-α). Through qPCR, western blot and confocal analysis, we demonstrated higher expression levels of PARP-14 in αTC1.6 cells with respect to βTC1 cells under inflammatory stimuli. By cytofluorimetric and caspase-3 assays, we showed the higher resistance of α cells compared to β cells to apoptosis induced by cytokines. Furthermore, the ability of PJ-34 to modulate the expression of the proteins involved in the survival pathway suggests a protective role of PARP-14. These data shed light on a poorly characterized function of PARP-14 in αTC1.6 cells in inflammatory contexts, widening the potential pharmacological applications of PARP inhibitors.

## Introduction

Poly(ADP-ribosyl)ation is a well-known post-translational modification of proteins, which uses NAD^+^ as a substrate. It is involved in cell proliferation and survival, DNA repair, inflammation and cell death (1–5). PARP-1 is the most studied member of the PARP super-family, which is comprised of 17 proteins in humans. The PARP family proteins are encoded by different genes, all displaying a conserved catalytic domain containing PAR (poly ADP ribose) (6–11). PARP-1, PARP-2, PARP-5a, and PARP-5b catalyze the polymerization of ADP-ribose units (PARylation) through α(1 → 2) O-glycosidic bonds in linear or branched chains (12). PARP mono enzymes, mono-(ADP-ribosyl) transferases (MARTs) (i.e., PARP-3, PARP-4, PARP-6, PARP-10, PARP-14, PARP-15, and PARP-16) catalyze the addition of a single ADP-ribose unit to target proteins through a process called MARylation (13). The implication of poly- or mono-(ADP-ribosyl)ation in many pathological conditions has promoted the development of pharmacological molecules able to block these processes (12, 14). PARP-14, also known as B aggressive lymphoma protein (BAL2 protein) in humans, belongs to macro-PARPs, a branch of the PARP family (15). Recent evidence has highlighted the role of PARP-14 in cancer and in many other diseases (15–20). In multiple myeloma, the involvement of PARP-14 in the JNK pathway has been reported, precisely between JNK2 and JNK1 (16). By binding to JNK1 and inhibiting its pro-apoptotic activity, PARP-14 acts as a pro-survival signal. This inhibition would be responsible for blocking the phosphorylation of downstream proteins such as p53, promoting cell survival (21). In our study, we used mouse αTC1.6 glucagonoma and βTC1 insulinoma cells as experimental models to verify the molecular basis of the involvement of PARP-14 in the JNK pathway in pancreatic inflammatory state. It is well-established that pro-inflammatory cytokines, such as interleukin-1β (IL-1β), interferon-γ (IFNγ) and tumor necrosis factor-α (TNFα), are major candidates for causing apoptotic death of pancreatic β cells and immune-mediated diabetes (20, 22–25). Therefore, we set out to investigate the ability of α cells, compared to β cells, to resist cytokines, evaluating the role played in this system by PARP-14, which we found to be overexpressed in αTC1.6 cells. The results obtained, also by using the PARP inhibitor PJ-34, allowed us to confirm that PARP-14 is involved in a transduction pathway mediated by JNKs and plays a protective role by promoting α cell survival.

## Materials and Methods

### Chemicals and Antibodies

Reagent grade chemicals were purchased from Sigma Chemicals Co. (St. Louis, MO, USA) or E. Merck (Darmstadt, Germany, EU). PARP-14 inhibitor PJ-34 [N-(6-Oxo-5,6-dihydrophenanthridin-2-yl)-N,N-dimethylacetamide.HCl] was from Calbiochem. (La Jolla, CA, USA). The primary antibodies against PARP-14 (mouse monoclonal and goat polyclonal antibodies) were from Santa Cruz Biotechnology Inc. (CA, USA); rabbit polyclonal antibody to c Jun N terminal Kinase 1 (JNK1) and rabbit polyclonal antibody to c Jun N terminal Kinase 2 (JNK2) were from Proteintech™; rabbit polyclonal antibody against phospho-p53 and mouse monoclonal antibody against p53 were purchased from Cell Signaling Technology® and mouse monoclonal antibody against Glyceraldehyde 3-phosphate dehydrogenase (GAPDH) was from Abcam. Reagents for qPCR Trizol, deoxyribonuclease 1 (DNase I Amplification Grade), High Capacity RNA-to-cDNA Kit, Power SYBR® Green PCR Master Mix, were from Lifetechnologies™, Foster-City (CA, USA).

### Cell Culture and Treatment With Cytokines

Mouse glucagonoma αTC1.6 cells were purchased from the American Type Culture Collection (ATCC). Cells were maintained in Dulbecco's Modified Eagle Medium (DMEM—Sigma-Aldrich, Saint Louis, MO, USA) containing 10% fetal bovine serum (FBS), 2 mM L-glutamine, 0.15% 4-(2-hydroxyethyl)-1-piperazineethanesulfonic acid (HEPES) 15 mM, 1% non-essential amino acids (NEAA), 0.02% bovine serum albumins (BSA, Sigma-Aldrich), 25 mM d-glucose (Sigma-Aldrich), 100 U/ml penicillin, and 100 μg/ml streptomycin, at 37°C, with 5%-CO_2_ humidified incubator. Mouse insulinoma βTC1 cells were also from ATCC; cells were cultured in DMEM with 25 mM glucose (Sigma Aldrich), supplemented with 2 mM L-Glutamine, 15% horse serum (HS), 2.5% FBS, 1% penicillin/streptomycin, in 95% humidified air, with 5% CO_2_ at 37°C. Cells were passaged once a week after trypsinization and replaced with new medium twice weekly. Both cell lines were treated with a cytokine cocktail (recombinant murine IL-1β, specific activity 0.1 U/ml, Peprotech, London, UK, UE; recombinant murine IFN-γ, specific activity 25 U/ml, Peprotech; recombinant murine TNF-α, specific activity 25 U/ml, Peprotech), as previously described (22).

### qPCR

Total RNA was extracted with TRIzol (Life Technologies, Foster City, CA, USA), according to the manufacturer's instructions. RNA quantification was performed by Qubit Fluorometer (Life Technologies). Contaminant DNA was removed using deoxyribonuclease 1 (DNase I Amplification Grade; Life Technologies). DNase-treated RNA was reverse transcribed by using a High Capacity RNA-to-cDNA Kit (Life Technologies), according to the manufacturer's instructions. Resulting cDNAs (30 ng per sample) were amplified through an ABI PRISM 7900HT Fast Real-Time PCR System (Life Technologies), as previously described (26). Single-gene specific assays were performed through real-time PCR by using Fast SYBR Green Master Mix (Life Technologies) according to the manufacturer's instruction. To allow statistical analysis, PCRs were performed in three independent biological replicates. The list of the primer pairs used for comparison analysis is reported below.

**Table:** 

**Gene Symbol**	**Forward**	**Reverse**
PARP1	CTCTCCAATCGCTTCTACAC	GTTGTCTAGCATCTCCACCT
PARP2	CTCCATCCCTCCAGTAATCC	ATTTCAATGTCTCCCAATGCC
PARP3	GAACCTTATCACCAACATCTTCAG	GGCATCTTCTTCACATCCAG
PARP4	GATAACAGCACACTTCCTCC	TTGTCTCTTCAGGTCTTCCAC
TNKS1	GGCATATCCAGAATATCTCATCAC	GGTCACTAGGTCTTCTGCTC
TNKS2	GCCAACCATCCGAAATACAG	CTTATAGTCACCAGTCAGAACAG
PARP6	AGCATCTTCTCACCCATTCC	GAACGATTCATAAGGCGTCC
PARP7	GAATGAAGTATGGAGGACAAGAC	GAGTTTGAATTACTACAGGGTGG
PARP8	TTCTGGATGAGGAGATTGCTG	ACTGGGCCGTTTAAATACTG
PARP9	GTACACATTTCAACGATACCC	CACCTTATTGTCTATCTGCTCC
PARP10	CTTGAAGGAACGGATGTGAC	GACTCTGAAGCAACTCTTGG
PARP11	AAGCAGATGAATCTTGTCAC	ACAGATGTAACTGAAGGCAC
PARP12	CCTGGGATTTAAGAAGATCACTC	AAAGTAGAAAGGTAGTGTACGG
PARP14	TGCCAAGCAGTCAGTGATGTC	CCTGGAAAACTGTGTGCTCTAT
PARP16	ACCTGAACAAGACTTCTCTG	CCATGAGGACTATAAATGAGGG
Trp53	CACGCTTCTCCGAAGACTGG	TCTTCTGGAGGAAGTAGTTTCCAT
Mapk8 (JNK1)	TCAAGCACCTTCACTCTGCTG	TCCTCGCCAGTCCAAAATCAA
Mapk9 (JNK2)	TACCAGATGCTCTGTGGCAT	TACAGGCTGTTCGCGCC
PPIA	CAGAGCCACTGTCGCTTT	TGTCTTTGGAACTTTGTCTGCAA
HPRT	CAGTCAACGGGGGACATAAA	GGGCTGTACTGCTTAACCAG

### Immunofluorescence

Murine pancreatic αTC1.6 and βTC1 cells were cultured on a sterile circular cover-glass (12 mm diameter, from Electron Microscopy Sciences, PA, USA), inserted in a 24-well plate. After incubation (24 and 48 h), with or without cytokines, cells were processed as previously reported (27). Pancreatic αTC1.6 and βTC1 cells were fixed with 3% paraformaldehyde in phosphate buffered saline (PBS) at 4°C for 30', washed twice with PBS for 5' and permeabilized with 0.2% TritonX-100 in PBS for 10'. Non-specific sites were blocked by incubation in 5% BSA at 20°C for 30'. Subsequently, both cell lines were incubated overnight at 4°C with the primary goat polyclonal antibody against PARP-14 (diluted 1:100 in PBS containing 1% BSA) in a moist chamber. Following three washing steps with PBS for 5', anti-goat FITC-conjugated secondary antibody (Santa Cruz), diluted 1:50 in PBS containing 1% BSA, was added for 1 h at 20°C in a dark chamber. Following the fluorescent labeling procedures, the slides were washed three times (5'each) with PBS, air dried, mounted up-side down on glass slides and covered with a drop of DAPI solution (Electron Microscopy Sciences) to counterstain the nucleus. Negative controls did not include both primary antibodies.

### Confocal Microscopy Imaging

For confocal imaging, we used an Olympus FV1000 confocal laser scanning microscope (LSM), equipped with Diode UV (405 nm, 50 mW), multiline Argon (457 nm, 488 nm, 515 nm, total 30 mW), HeNe (543 nm, 1 mW), and HeNe(R) (633 nm, 1 mW) lasers. An oil immersion objective (60x O PLAPO) and spectral filtering system were used. The detector gain was fixed at a constant value; images were acquired at random locations throughout the area in sequential mode. Quantitative analysis of fluorescence was performed by using the ImageJ software (1.50i version, NIH), in terms of the average of the mean gray value measured at least in 10 equivalent regions of interest (ROIs), drawn in the cell-covered areas. The values obtained by these analysis were imported into the OriginPro 8.6 program for statistical analysis for *p*-values, calculated by using a one-way ANOVA with a Tukey multiple comparison test.

### Caspase-3 Colorimetric Protease Assay

Caspase-3 activity was evaluated on αTC1.6 and βTC1 cell lysates through a colorimetric protease assay (Thermo Fisher Scientific), following the manufacturer's protocol. Briefly, cells (αTC1.6 and βTC1) were seeded at a concentration of 5 × 10^6^ cells in 100 mm petri dish for each experimental condition [CTRL; 10 μM PJ-34; cytokines (CYT: TNF-α 25 U/ml; IFN-γ 25 U/ml and IL-1ß 0.1 U/ml); CYT + 10 μM PJ-34]. After 24 and 48 h of incubation the cells were lysed and centrifuged at 10,000 g for 1 min at 4°C. The supernatant containing 100 μg of total protein were incubated with 5 μL caspase substrate in the 50 μL reaction buffer at 37°C for 2 h in the dark. The caspase-3 activity was determined by a microplate reader (Synergy 2-bioTek) set at 400 nm.

### Imaging Flow Cytometer Analysis

αTC1.6 and βTC1 cells were seeded in 6-well plates at a density of 3 × 10^4^ cells. After incubation for 16 h, the two cell lines were exposed to the treatments. At the appropriate time points, cells were collected, washed with PBS and stained with Annexin V-FITC/Propidium Iodide (PI), in Annexin-V binding buffer (Sigma-Aldrich), according to the manufacturer's instructions. Cells were incubated for 10' at 20°C and protected from light. Samples were analyzed immediately by flow cytometer FlowSight® (Amnis®, part of EMD Millipore) as previously reported (28). A 488 nm laser was used for excitation. Bright field (430–480 nm), Annexin V-FITC (505–560 nm) and PI (595–642 nm) analysis were focused on at least 5.000 cell events per sample. INSPIRE® software (http://www.merckmillipore.com) was used to setup, calibrate and obtain spectral compensation, while IDEAS® [version 6.0 software (http://www.merckmillipore.com)] was used to quantify the numbers of vital (Annexin V and PI negative, double negative), early apoptotic (Annexin V positive/PI negative), late apoptotic (Annexin V and PI positive, double positive) and necrotic cells (PI positive). The distribution of acquired events in the scatter plot, depending on their differential fluorophore labeling, is shown in the results section.

### Western Blot

The expression of PARP-14, JNK1 and JNK2, and the level of phospho-p53 were evaluated by western analysis. Pancreatic αTC1.6 and βTC1 cells were grown for 24 and 48 h with normal medium (control) or stimulated by a cytokine cocktail, either in the presence or in the absence of 10 μM PJ-34 inhibitor (added simultaneously). Cells were lysed as previously described (29, 30). Cell lysate proteins were quantified with a bicinchoninic acid (BCA) protein assay kit (Pierce™, ThermoFisher Scientific). Immunoblots (30 μg cell lysate proteins) were performed as described elsewhere (29). Membranes were incubated with primary antibodies against PARP-14 (mouse monoclonal antibody, 1:500 dilution), JNK1 (rabbit polyclonal antibody, 1:5000 dilution), JNK2 (rabbit polyclonal, 1:4000), phospho-p53 (rabbit polyclonal antibody, 1:1000 dilution) and total p53 (mouse monoclonal antibody 1:1000). Membranes were then incubated with secondary antibodies for 1 h at 20°C and immune complexes were detected by an enhanced chemiluminescence reagent (ECL, Amersham). Relative phosphorylation or protein levels were quantified by using the ImageJ program. Immunoblots were normalized through GAPDH mouse monoclonal antibody (1:2000 dilution).

### Statistical Analysis

Data are expressed as mean ± standard deviation (S.D.) of three independent experiments (i.e., biological and technical triplicates). We evaluated the statistical significance of these data by applying Student's *t*-test or one-way Anova test, as described in figure legends.

## Results

### PARP Family Expression in Pancreatic αTC1.6 and ßTC1 Cells Treated With Inflammatory Cytokines for 24 and 48 h

By qPCR, we verified if any member of the PARP family was differentially expressed (DE) in pancreatic αTC1.6 and βTC1 cell lines, in the presence of the following cytokine concentrations: TNF-α 25 U/ml; IFN-γ 25 U/ml and IL-1ß 0.1 U/ml, compared with the steady state, at 24 h (Table S1) and 48 h (Table 1). These cytokine concentrations were chosen after dose-response and time-course experiments, evaluated by MTT and FACS analysis (data not shown). In the presence of an inflammatory environment, significant fold change values were found for many PARPs, in αTC1.6 and βTC1, at both 24 h (Table S1) and 48 h (Table 1). Notwithstanding, PARP-14 was the only one of the PARP family that was significantly differentially expressed between the two cell lines (Table 2). The high expression levels of PARP-14 in αTC1.6 cells, compared with those obtained in βTC1 cells, allowed us to hypothesize a potentially important role of PARP-14 in αTC1.6 cells. As a valid reference model to study cell survival in an inflammatory state, we selected βTC1 cells, since they are very susceptible to the action of cytokines.

**Table 1:** Fold change values of 15 PARP family members in murine pancreatic αTC1.6 and βTC1 cells after 48 h of cytokine treatment.

**PARP family member**	**Avg FC αTC1.6 CYT 48 h**	**Std dev**	***p*-values**	**Avg FC βTC1 CYT 48 h**	**Std dev**	***p*-values**
Parp1	0.98	±0.39	0.7123734	0.67	±0.21	0.8364281
Parp2	0.98	±0.36	0.9890045	0.82	±0.34	0.8416218
Parp3	5.19	±1.68	0.0265288	6.05	±1.23	0.0046319
Parp4	1.27	±0.74	0.7370967	1.41	±0.07	0.0063723
Tnks	0.82	±0.42	0.6121232	0.81	±0.30	0.9541871
Tnks2	1.02	±0.42	0.8511544	1.23	±0.06	0.0440711
Parp6	1	±0.27	0.8383906	1.11	±0.29	0.3679152
Parp7	1.02	±0.22	0.4051082	0.65	±0.14	0.3227707
Parp8	1.38	±0.62	0.8922677	1.21	±0.21	0.0924684
Parp9	27.88	±1.71	0.0000092	35.48	±0.56	0.0000007
Parp10	3.95	±0.55	0.0035363	3.83	±1.42	0.0305762
Parp11	3.28	±0.16	0.0000512	5.43	±0.68	0.0002361
Parp12	4.27	±0.99	0.0142340	2.61	±0.71	0.0208804
Parp14	2102.5	±13.10	0.0000002	122.48	±0.91	0.0000001
Parp16	1.62	±0.55	0.2686825	1.66	±0.33	0.0345925

*Fold change values (Avg FC) of each PARP are reported comparing PARP ΔCt values of the cells treated with the cytokine (CYT) cocktail (TNF-α 25 U/ml; IFN-γ 25 U/ml and IL-1β 0.1 U/ml) and PARP ΔCt values of steady state cells (Control: CTRL), at 48 h. qPCR experiments were carried out in triplicate (n = 3). Statistical significance was determined with Student's t-test. Std Dev and p-value columns indicate the standard deviation values and the significance of every single PARP, respectively*.

**Table 2:** Expression profile of 15 candidate PARPs in αTC1.6 and βTC1 after treatment with cytokines for 48 h.

**PARP family member**	**Avg ΔΔCt αTC1.6**	**Avg ΔΔCt βTC1**	***p*-value**
Parp1	−0.15	0.07	0.66
Parp2	0.01	−0.08	0.87
Parp3	−2.10	−2.68	0.21
Parp4	−0.24	−0.59	0.60
Tnks	0.29	0.02	0.67
Tnks2	0.09	−0.22	0.51
Parp6	−0.06	−0.27	0.57
Parp7	0.23	0.26	0.90
Parp8	−0.08	−0.47	0.51
Parp9	−2.75	−2.49	0.55
Parp10	−2.23	−1.96	0.50
Parp11	−0.37	−1.02	0.10
Parp12	−1.94	−1.62	0.40
Parp14	−8.88	−4.53	0.01
Parp16	−0.53	−0.92	0.38

*Expression values are reported as ΔΔCt, according to a color variation from green to red. Higher ΔΔCts (in green) correspond to a lower PARP expression instead, lower ΔΔCts (in red) correspond to a higher PARP expression. PARP14 induction, in bold, is significantly higher in αTC1.6 with respect to βTC1 (p = 0.01, n = 3, Student's t-test)*.

### mRNA Expression of PARP-14 in Pancreatic αTC1.6 and ßTC1 Cells, Following 24 and 48 h of Cytokine Treatment

**Figure 1:**
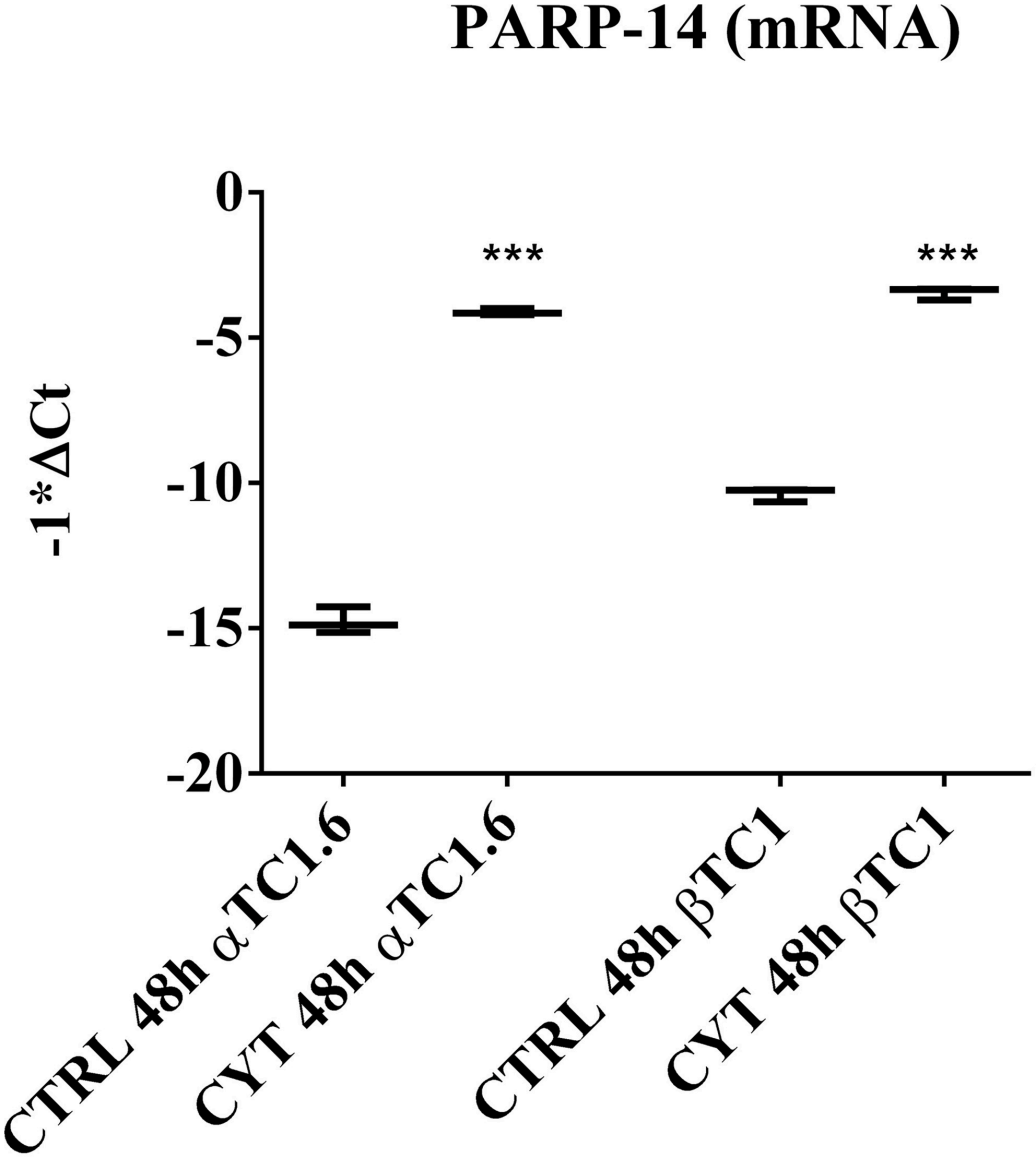
PARP-14 mRNA expression in murine pancreatic αTC1.6 and βTC1 cells following 48 h of cytokine treatment. Pancreatic αTC1.6 and βTC1 cells were grown in normal medium (Control: CTRL) or in the presence of cytokine cocktail (CYT: TNF-α 25 U/ml; IFN-γ 25 U/ml and IL-1β 0.1 U/ml), for 48 h. Box and whisker plots represent PARP-14 mRNA expression levels in αTC1.6 and βTC1 cells exposed to inflammatory stimuli compared to their relative control. Y-axis represents the distribution of −1*ΔCt values for PARP-14 mRNA. The qPCR experiments were carried out in triplicate (*n* = 3). Statistical significance was determined with Student's *t*-test, comparing the control ΔCt values (CTRL) to those of cytokine-treated samples (CYT). Asterisks represent a significant difference between the CYT and CTRL (^***^p < 0.001).

The box plots in Figure 1 show the different expression levels of PARP-14 mRNA between the two pancreatic cell phenotypes, after treatment with cytokines at 48 h (Figure 1). According to the data shown in Tables 1, 2, the inflammatory state induced a significant increase of PARP-14 mRNA expression levels in both cell lines, though this increment was significantly higher in αTC1.6 cells. A similar trend was observed in the same experimental conditions at 24 h (Figure S1).

### PARP-14 Protein Expression in Pancreatic αTC1.6 and ßTC1, Following 24 and 48 h of Cytokine Treatment: Confocal Microscopy Analysis

**Figure 2:**
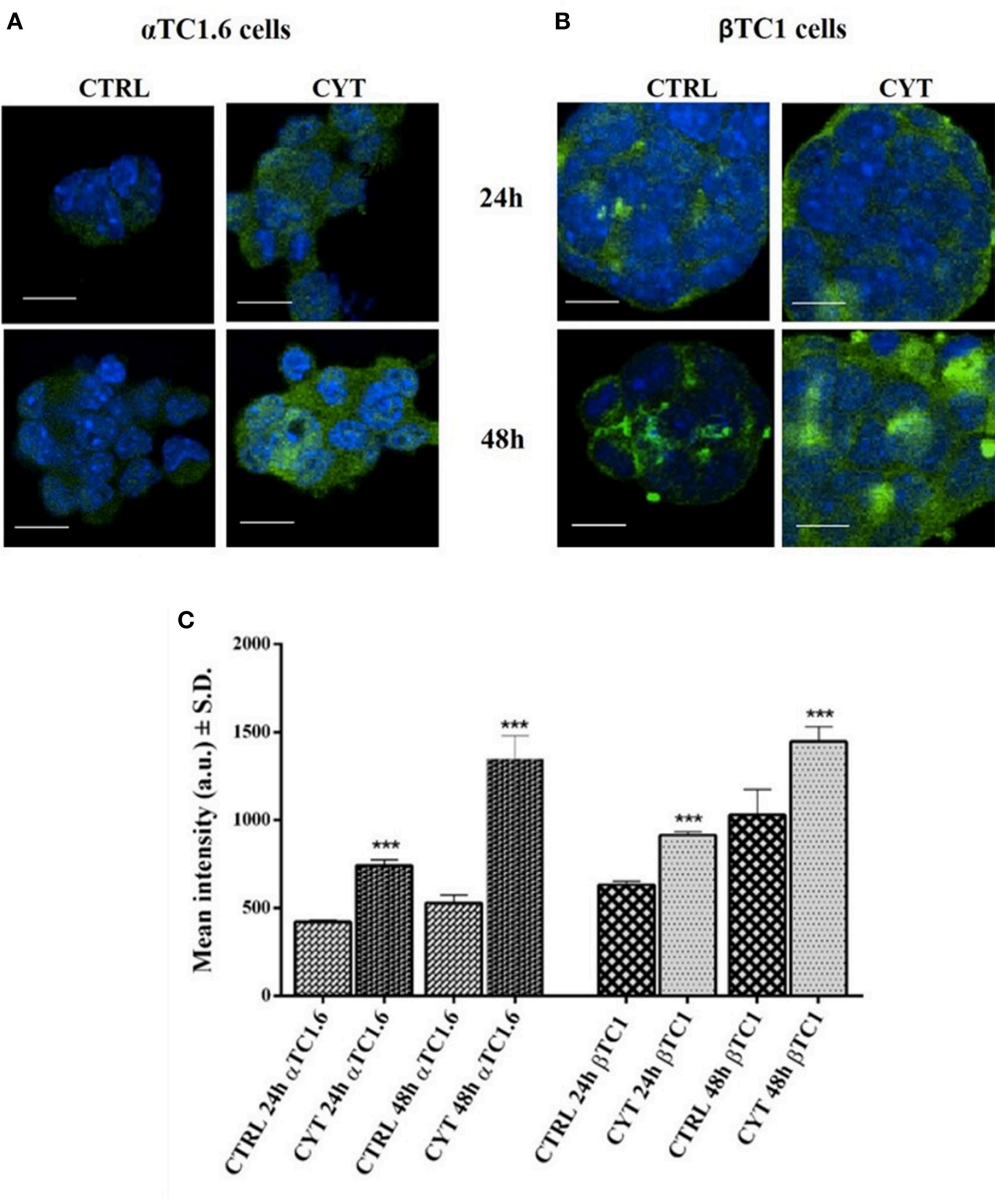
Confocal LSM of PARP-14 expression in pancreatic αTC1.6 and βTC1 cells, following 24 and 48 h of cytokine treatment. Confocal microscopy of PARP-14 expression in pancreatic αTC1.6 **(A)** and βTC1 cells **(B)**. The two cell lines were cultured in normal medium (Control: CTRL) or in medium containing cytokines (CYT: TNF-α 25 U/ml; IFN-γ 25 U/ml, and IL-1β 0.1 U/ml) for 48 h. Cells were stained with a polyclonal anti-goat FITC-conjugated secondary antibody. Green fluorescence represents the distribution of PARP-14 inside the cells. The blue fluorescence is due to the labeling with DAPI to mark the nuclei. The images were recorded at the following conditions of excitation/emission wavelengths: 405/425–475 nm (blue); 488/500–540 nm (green). Magnification x60; Scale bar = 20 μm. Quantitative analysis of Confocal LSM data **(C)**. The graphs show mean intensity values (a.u.) of PARP-14 fluorescence as measured on the confocal LSM ± SD (S.D. = standard deviation). Student's *t*-test was performed by using the data from 4 to 6 randomly chosen fields and a minimum of 10 cells in each field of the control sample (CTRL) compared to those of cytokines (CYT). All experiments were repeated 3 times (*n* = 3). Asterisks represent a significant difference between the CYT and CTRL (^***^p < 0.001).

The expression of PARP-14 in murine pancreatic αTC1.6 and ßTC1 cells treated with or without cytokines (TNF-α 25 U/ml; IFN-γ 25 U/ml and IL-1ß 0.1 U/ml) for 24 and 48 h, was analyzed through laser scanning confocal microscopy analysis (Figure 2). By using a green fluorescently-labeled antibody (FITC secondary antibody), we analyzed PARP-14 immunofluorescence in αTC1.6 and ßTC1 cells, grown for 24 and 48 h in normal culture medium (controls) or in the presence of inflammatory cytokines, at the concentrations mentioned above (Figures 2A,B). In αTC1.6 cells, the treatment with cytokines induced a significant increase of the PARP-14 immunofluorescence signal, compared with the control, mainly at 48 h (Figure 2A). However, in ßTC1 cells the PARP-14 immunofluorescence signal was higher in the presence of cytokines and the basal level appears more evident than αTC1.6, especially at 48 h (Figure 2B). Therefore, despite the increment of PARP-14 immunofluorescence in both cell lines, this protein was more overexpressed in αTC1.6 than ßTC1 cells, particularly at 48 h (Figures 2A,B). Quantitative analysis of confocal micrographs was carried out to analyze the fluorescence recorded for the FITC secondary antibodies (Figure 2C). In both cell types, there was a statistically significant increase of the fluorescence intensity for PARP-14 after cytokine treatment, however, at 48 h, in αTC1.6 cells, the intensity almost doubled that measured at 24 h, compared to that measured for ßTC1 cells.

### Caspase-3 Activity in Pancreatic αTC1.6 and ßTC1 Cells, Following 24 and 48 h of Cytokine Treatment, in the Presence or Absence of PJ-34

**Figure 3:**
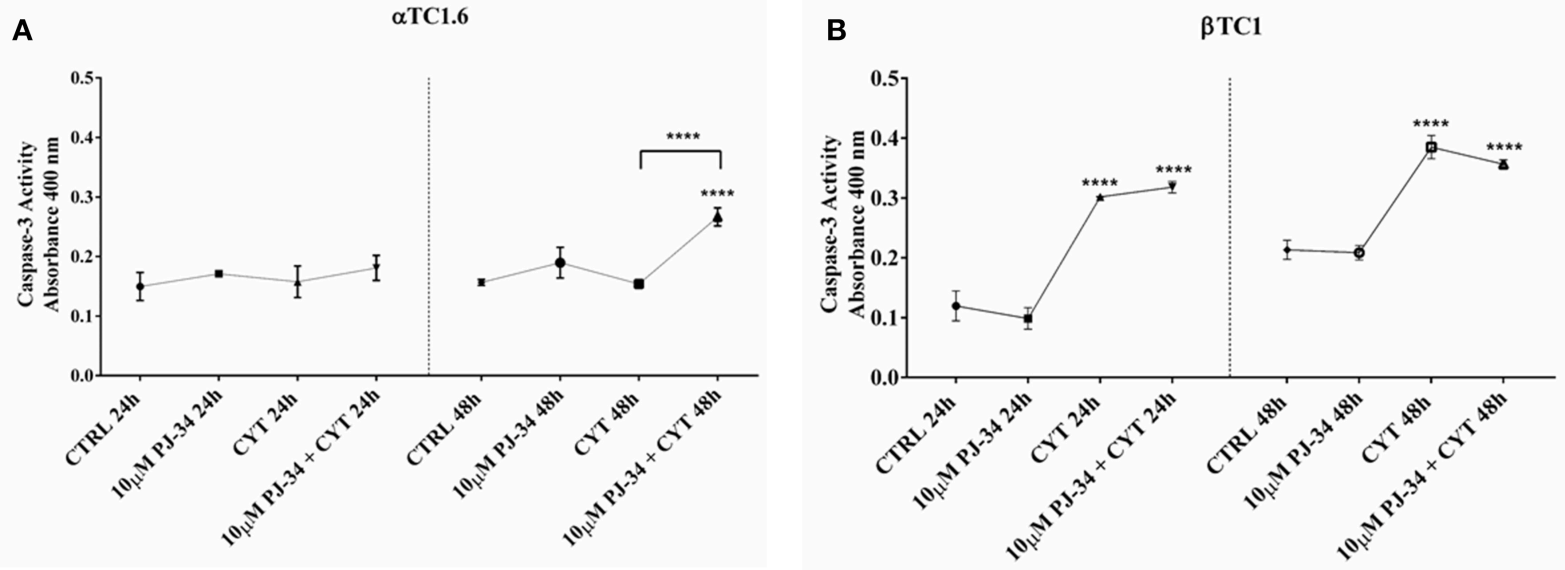
Caspase-3 activity of αTC1.6 and βTC1 cells following 24 and 48 h of cytokine treatment, in the presence or absence of 10 μM PJ-34. Pancreatic αTC1.6 **(A)** and βTC1 cells **(B)** were cultured in full medium with (CYT) or without (Control: CTRL) cytokine cocktail (TNF-α 25 U/ml; IFN-γ 25 U/ml, IL-1β 0.1 U/ml), both in the presence or absence of 10 μM PJ-34, for 24 and 48 h. Caspase-3 activity was evaluated through a colorimetric protease assay, as described in the Materials and Methods section. In Y-axis are reported the means ± SD of absorbance values of each experimental condition (X-axis) at 24 and 48 h (S.D. = standard deviation). All experiments were repeated 3 times (*n* = 3). Statistical analysis was performed by One-way Anova test, using control (CTRL) and cytokines (CYT) conditions as reference sample. Asterisks represent a significant difference between the treated samples and CTRL. The significance between CYT +10 μM PJ-34 and CYT is indicated by the asterisks upon the sticks (^****^p < 0.0001).

Caspase-3 assay was performed on pancreatic αTC1.6 and ßTC1 cell lines to evaluate apoptosis induction by the cytokine cocktail. Furthermore, we also tested the effects of the PARP inhibitor PJ-34 on the biomolecular functions of PARP-14. The graphs in Figure 3 show the caspase-3 activity of αTC1.6 (Figure 3A) and ßTC1 (Figure 3B), treated with cytokines (TNF-α 25 U/ml; IFN-γ 25 U/ml and IL-1ß 0.1 U/ml), in the presence or absence of 10 μM PJ-34, at 24 and 48 h. Unlike βTC1 cells, cytokine treatment of αTC1.6 did not cause significant changes in the caspase-3 activity, at both 24 and 48 h (Figures 3A,B). No variation of the caspase-3 activity was observed when 10 μM PJ-34 was added, simultaneously, to the cytokines, at 24 h, in both cell lines (Figures 3A,B). However, at 48 h, the addition of PJ-34 to the cytokines produced a different result in the two cell lines. In fact, while in ßTC1 cells the addition of the PARP inhibitor did not produce any significant effects on the caspase-3 activity, in αTC1.6 cells the presence of PJ-34 caused a significant increase of the enzymatic activity compared to the cytokines alone (Figure 3A). This result could suggest a protective role of PARP-14 in αTC1.6.

### Evaluation of Apoptotic Death on Pancreatic αTC1.6 and ßTC1 Cells Grown With Cytokines, in the Presence or Absence of 10 μM PJ-34: Flow Cytometry Assay

**Figure 4:**
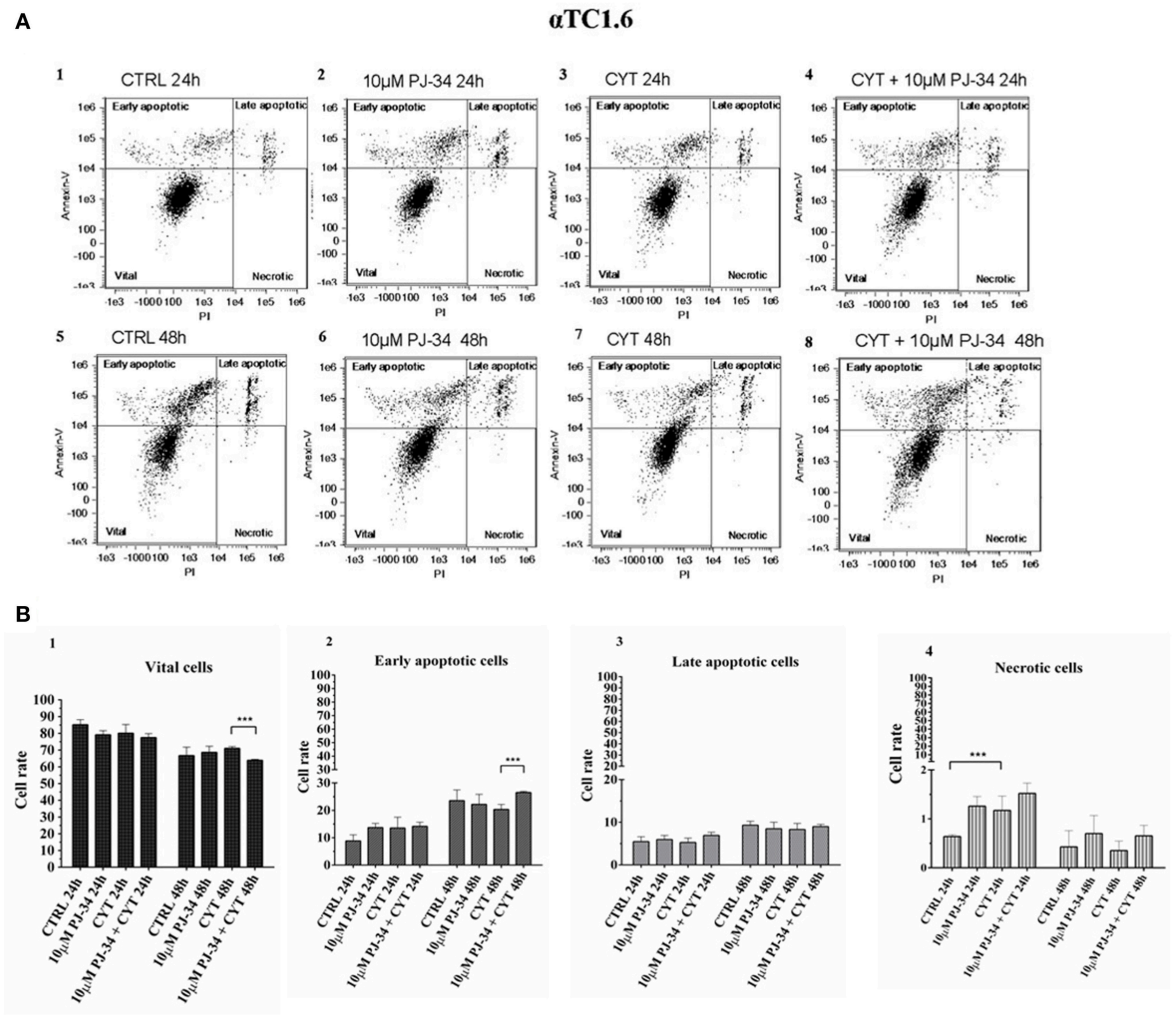
Evaluation of apoptosis in αTC1.6 cells, grown for 24 and 48 h in the presence or absence of cytokines, with or without 10 μM PJ-34 by Annexin V/PI assay: flow cytometry. **(A)** Flow cytometry scatter plots of αTC1.6 cells, grown, for 24 and 48 h, in the following conditions: in normal medium (CTRL; plots 1 and 5); in the presence of 10 μM PJ-34 (plots 2 and 6); in the presence of cytokine cocktail (CYT; plots 3 and 7); in the presence of cytokines and 10 μM PJ-34 (CYT + 10 μM PJ-34; plots 4 and 8). Each plot, representing a single experimental condition, is divided into four squares, in which the cells are distributed based on their Annexin-V and Propidium Iodate (PI) positivity and/or negativity. **(B)** 1–4. The histograms show the percentage of the single cell subpopulations: vital cells, early apoptotic cells; late apoptotic cells; necrotic cells, for each experimental condition (CTRL; 10 μM PJ-34; CYT; CYT + 10 μM PJ-34), at 24 and 48 h. Statistical analysis was made using One-way Anova test, using control (CTRL) and cytokines (CYT) conditions as reference samples. The bars represent means ± SD of three independent experiments (*n* = 3; S.D. = standard deviation). Asterisks represent a significant difference between the treated samples and CTRL. The significance between CYT +10 μM PJ-34 and CYT is indicated by the asterisks upon the sticks (^***^p < 0.001).

**Figure 5:**
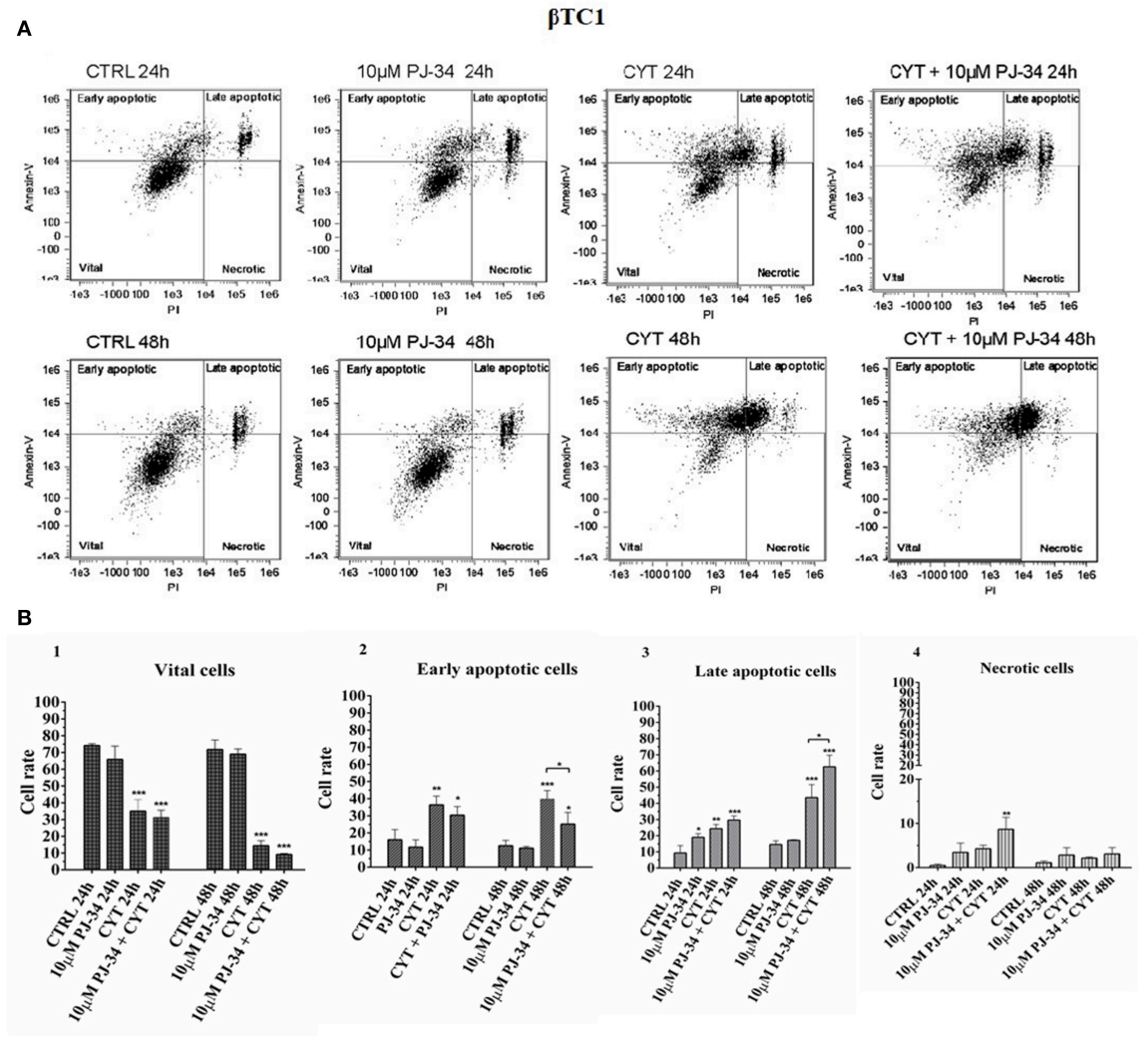
Evaluation of apoptosis in βTC1 cells grown for 24 and 48 h in the presence or absence of cytokines, with or without 10 μM PJ-34 by Annexin V/PI assay: flow cytometry. **(A)** Flow cytometry scatter plots of βTC1 cells, grown for 24 and 48 h in the following conditions: in normal medium (CTRL; plots 1 and 5); in the presence of 10 μM PJ-34 (plots 2 and 6); in the presence of cytokine cocktail (CYT; plots 3 and 7); in the presence of cytokines and 10 μM PJ-34 (CYT + 10 μM PJ-34; plots 4 and 8). Each plot, representing a single experimental condition, is divided into four squares, in which the cells are distributed, based on their Annexin-V and Propidium Iodate (PI) positivity and/or negativity. **(B)** 1–4. The histograms show the percentage of the single cell subpopulations: vital cells; early apoptotic cells; late apoptotic cells; necrotic cells, for each experimental condition (CTRL; 10 μM PJ-34; CYT; CYT + 10 μM PJ-34), at 24 and 48 h. Statistical analysis was made using One-way Anova test, using control (CTRL) and cytokines (CYT) conditions as reference samples. The bars represent means ± SD of three independent experiments (*n* = 3; S.D. = standard deviation). Asterisks represent a significant difference between the treated samples and CTRL. The significance between CYT +10 μM PJ-34 and CYT is indicated by the asterisks upon the sticks (^***^p < 0.001; ^**^p < 0.01; ^*^p < 0.05).

To verify the potential protective function of PARP-14 in this system, flow cytometry analysis was carried out on αTC1.6 and ßTC1 cells, grown in the presence or the absence of 10 μM PJ-34. This technique analyzes the percentage of vital, early/late apoptotic and necrotic cells, for each experimental condition [i.e., control, 10 μM PJ-34, cytokines (TNF-α 25 U/ml; IFN-γ 25 U/ml and IL-1ß 0.1 U/ml) and cytokines + 10 μM PJ-34]. Representative data of the Annexin V-FITC/Propidium Iodide (PI) flow cytometry experiments are shown in Figures 4A,B, 5A,B.

### Effect of PJ-34 on Apoptotic αTC1.6 Cell Death, Following 24 and 48 h of Cytokine Treatment

Each scatter plot shown in Figure 4A represents the distribution, in four squares, of pancreatic αTC1.6 cells according to their staining with Annexin-V and PI. At both 24 and 48 h the distribution of αTC1.6 cells was similar in all experimental conditions, indicating the resistance of these cells to apoptosis induction by inflammatory cytokines (Figure 4A, 1–8). The histograms shown in Figure 4B, 1–4, show the percentage of each cell subpopulation (vital, early/late apoptotic, necrotic) in the experimental conditions. It was interesting to note that cytokine treatment did not significantly affect αTC1.6 cell survival (Figure 4B, 1–4). However, only at 24 h, in the presence of cytokines, was a significant increment of necrotic cell rate, compared with the control, observed. However, the percentage of the necrotic cell subpopulation was under 2% of the total cells, in all the experimental conditions and at both time points. Furthermore, the concomitant presence of both PJ-34 and cytokines for 48 h caused a significant reduction of vital cells and a significant increase of the number of early apoptotic cells. This result convincingly suggests that the inhibition of PARP-14 makes pancreatic αTC1.6 cells susceptible to an inflammatory environment.

### Effect of PJ-34 on Apoptotic ßTC1 Cell Death, Following 24 and 48 h of Cytokine Treatment

The distribution of the ßTC1 cell population shown in the scatter plots, under inflammatory stimuli, appears very different with respect to αTC1.6 (Figure 5A, 1–8). In fact, when ßTC1 were grown in the presence of cytokines alone or in combination with PJ-34, most of these cells were scattered within the upper quadrants, proving that they were undergoing apoptosis, mainly at 48 h. The histograms in Figure 5B, 1–4 show the percentage of ßTC1 cell subpopulations (vital, early/late apoptotic and necrotic cells) shown in the scatter plots (Figure 5A, 1–8). It is evident that the inflammatory cytokines caused a sudden and drastic reduction of vital cells and a concomitant increase of early/late apoptotic and necrotic cells, independently of the addition of the PARP inhibitor, at both time points. This confirms the susceptibility of beta cells to inflammation.

### Evaluation of PAR-14, JNK1, and JNK2 Expression and p53 Phosphorylation in Pancreatic αTC1.6 and ßTC1 Cells Grown, for 24 and 48 h, With Cytokines, in the Presence or Absence of 10 μM PJ-34: Western Blot Analysis

To examine the potential signaling involvement of PARP-14 in a survival transduction pathway, mediated by c Jun N-terminal kinases 1 and 2 (JNK1/2), we performed a series of qPCR and western analysis on αTC1.6 and ßTC1 cells, grown in the presence or the absence of cytokines, with or without PJ-34, for 24 and 48 h. Furthermore, we also studied the activation of one of the most common apoptosis mediators, the phosphorylated p53 protein.

### Effect of the PARP Inhibitor PJ-34 on PARP-14 Expression in αTC1.6 and ßTC1 Cells, Grown for 24 and 48 h in the Presence or Absence of Cytokines

**Figure 6:**
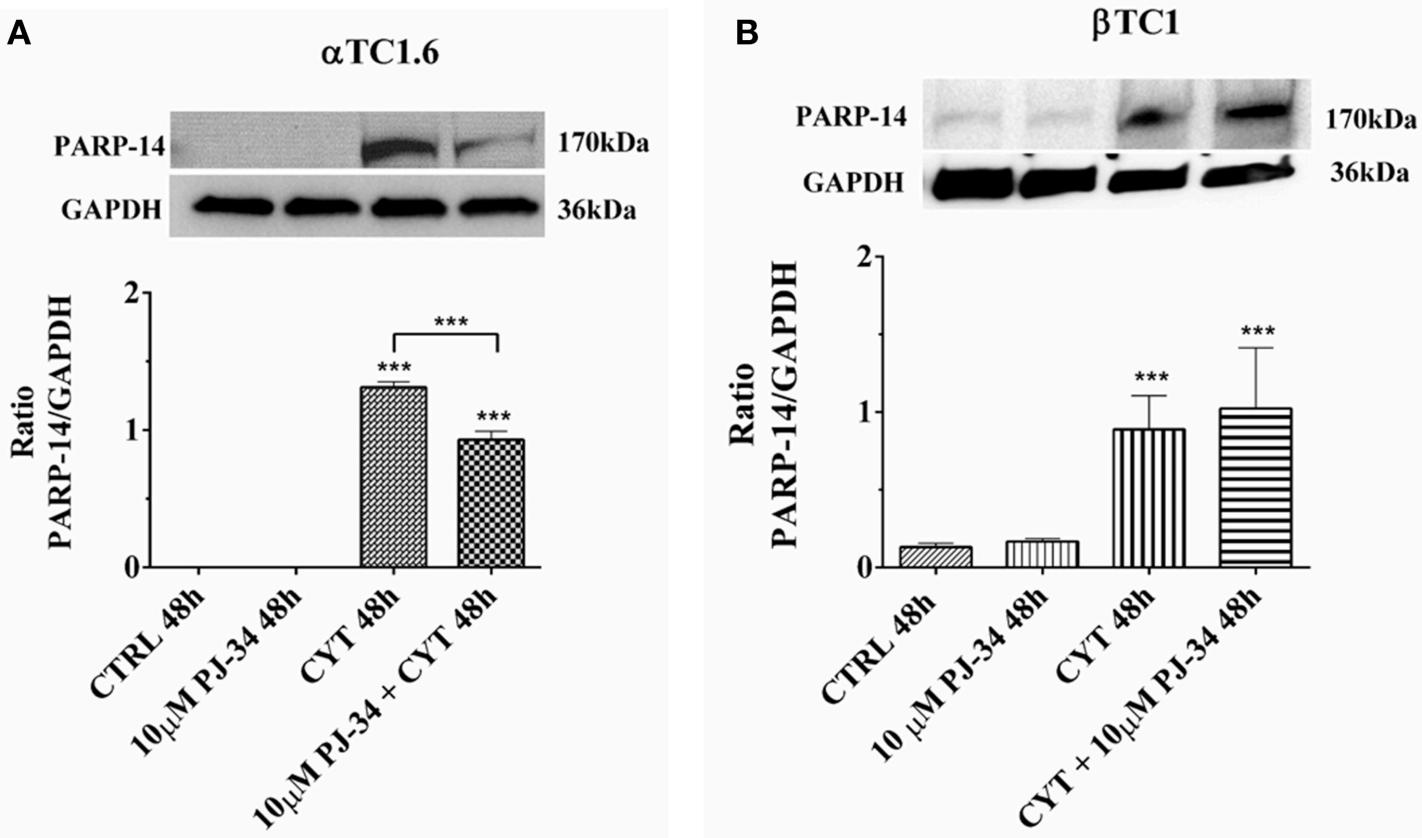
Effect of the PARP inhibitor PJ-34 on PARP-14 expression in αTC1.6 and βTC1 cells, grown for 48 h in the presence or absence of cytokines. αTC1.6 **(A)** and βTC1 **(B)** cells were grown in normal culture medium: control (CTRL); in the presence of 10 μM PJ-34; in culture medium containing cytokine cocktail (CYT: TNF-α 25 U/ml; IFN-γ 25 U/ml, and IL-1β 0.1 U/ml); in culture medium with the addition of both cytokine cocktail and 10 μM PJ-34 (CYT + 10 μM PJ-34), for 48 h. Expressed protein was revealed with a mouse monoclonal antibody against PARP-14 (1:500 dilution) as described in Materials and Methods section. The blots were controlled for equal loading by GAPDH, using a mouse monoclonal antibody (1:2000 dilution). Immunoreactive bands were visualized by chemiluminescence (ECL system).The values were obtained by the reading of blots using the Image J program. Statistical analysis was made using One-way Anova test, using control (CTRL) and cytokines (CYT) conditions as reference samples. The bars represent means ± SD of three independent experiments (S.D. = standard deviation). Asterisks represent a significant difference between the treated samples and CTRL. The significance between CYT +10 μM PJ-34 and CYT is indicated by the asterisks upon the sticks (^***^p < 0.001).

As a first approach, we verified how the inhibitor PJ-34 modulates the expression of PARP-14 both in αTC1.6 and βTC1 cells in our experimental conditions (Figures 6A,B and Figures S2A,B). The expression of PARP-14 in αTC1.6 cells was not detectable at the basal level (control and PJ-34 alone), at 24 h (Figure S2A) as well as 48 h (Figure 6A). On the other hand, cytokine exposition caused a significant increase of PARP-14 expression, mainly at 48 h (Figure 6A). At the same time point, the addition of PJ-34 to the cytokines significantly reduced PARP-14 expression (Figure 6A). In βTC1 cells, the increase of the protein level observed in an inflammatory environment was not reversed by the addition of PJ-34, both at 24 h (Figure S2B) and 48 h (Figure 6B).

### Effect of the PARP Inhibitor PJ-34 on JNK1 mRNA and Protein Expression in αTC1.6 Cells, Grown for 24 and 48 h in the Presence or Absence of Cytokines

**Figure 7:**
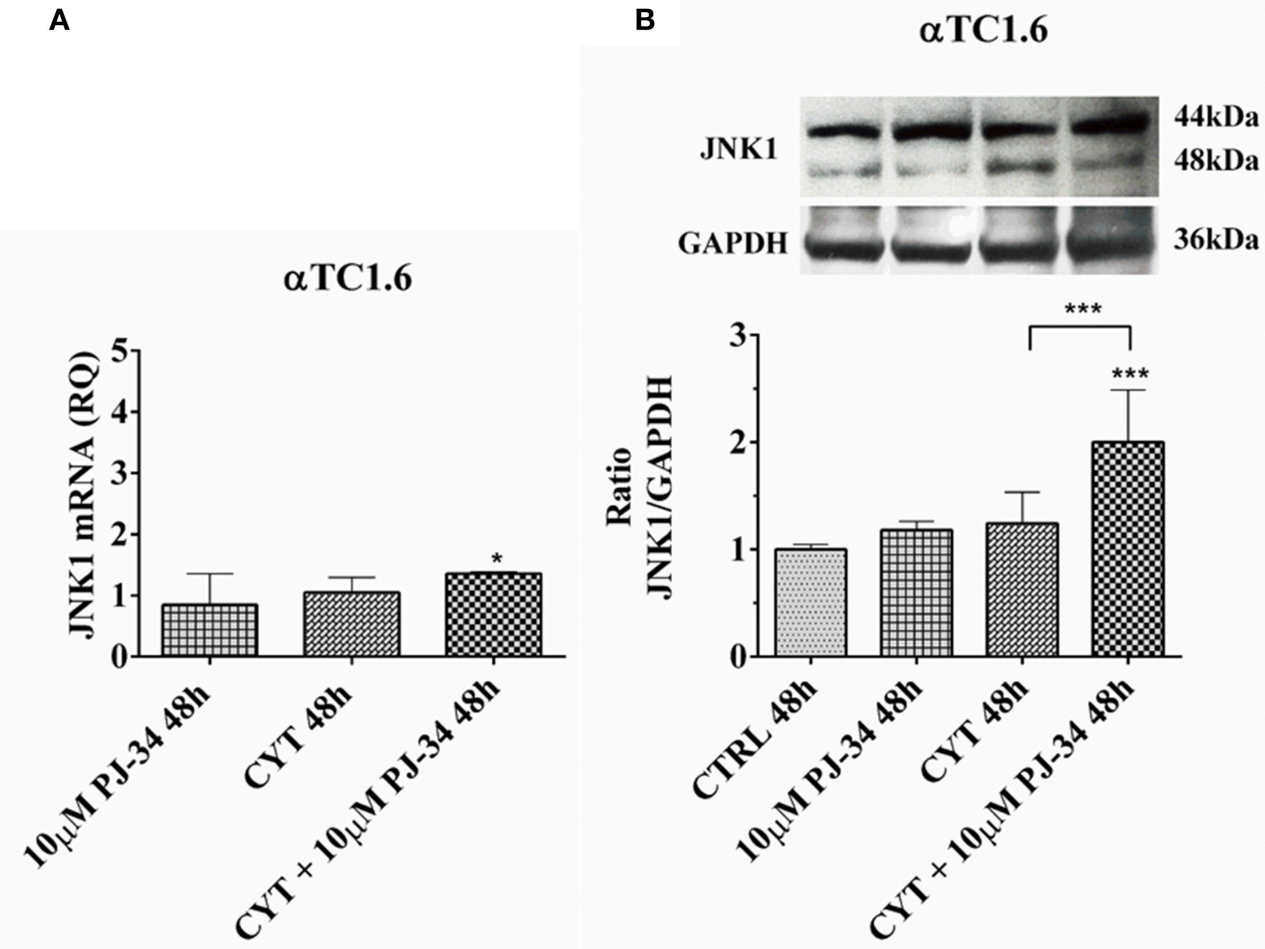
Effect of the PARP inhibitor PJ-34 on JNK1 mRNA and protein expression in αTC1.6 cells, grown for 48 h in the presence or absence of cytokines. Real-time PCR and total cell lysate immunoblottings were performed as described in the Materials and Methods section. αTC1.6 cells were grown: in normal culture medium (control: CTRL); in the presence of 10 μM PJ-34; in culture medium containing cytokine cocktail (CYT: TNF-α 25 U/ml; IFN-γ 25 U/ml and IL-1β 0.1 U/ml); in culture medium with the addition of both cytokine cocktail and 10 μM PJ-34 (CYT + 10 μM PJ-34), for 48 h. **(A)** Relative quantity (RQ) level of JNK1 mRNA, at 48 h, in the experimental conditions mentioned above. Relative quantification is referred to untreated cells. **(B)** JNK1 protein was revealed with a rabbit polyclonal antibody (1:5000 dilution) as described in Materials and Methods section. The blots were controlled for equal loading by GAPDH, using a mouse monoclonal antibody (1:2000 dilution). Immunoreactive bands were visualized by chemiluminescence (ECL system). The values were obtained by the reading of blots through the Image J program. Statistical analysis was carried out by One-way Anova test, using control (CTRL) and cytokines (CYT) conditions as reference samples. The bars represent means ± SD of three independent experiments (S.D. = standard deviation). Asterisks represent a significant difference between the treated samples and CTRL. The significance between CYT +10 μM PJ-34 and CYT is indicated by the asterisks upon the sticks (^***^p < 0.001; ^*^p < 0.05).

Since JNK1 is a pro-apoptotic molecule, activated by inflammatory signals, we wondered what the behavior of this protein in our experimental model could be. The trend of JNK1 mRNA was close to that of its encoded protein at both 24 h (Figures S3A,B) and 48 h (Figures 7A,B). After 24 h of treatment with cytokines, there was no significant reduction of the JNK1 mRNA levels, instead the JNK1 protein expression levels appeared significantly reduced vs. control (Figures S3A,B). This indicates the resistance of these cells to inflammatory insults. At the same time point, the addition of 10 μM PJ-34 to the cytokines did not produce any significant effect on both mRNA and protein levels (Figures S3A,B). Conversely, at 48 h, the addition of the inhibitor PJ-34 to the cytokines up-regulated JNK1 mRNA compared with the control and protein expression compared with the control and cytokines alone (Figures 7A,B).

### Effect of the PARP Inhibitor PJ-34 on JNK1 mRNA and Protein Expression in βTC1 Cells, Grown for 24 and 48 h in the Presence or Absence of Cytokines

**Figure 8:**
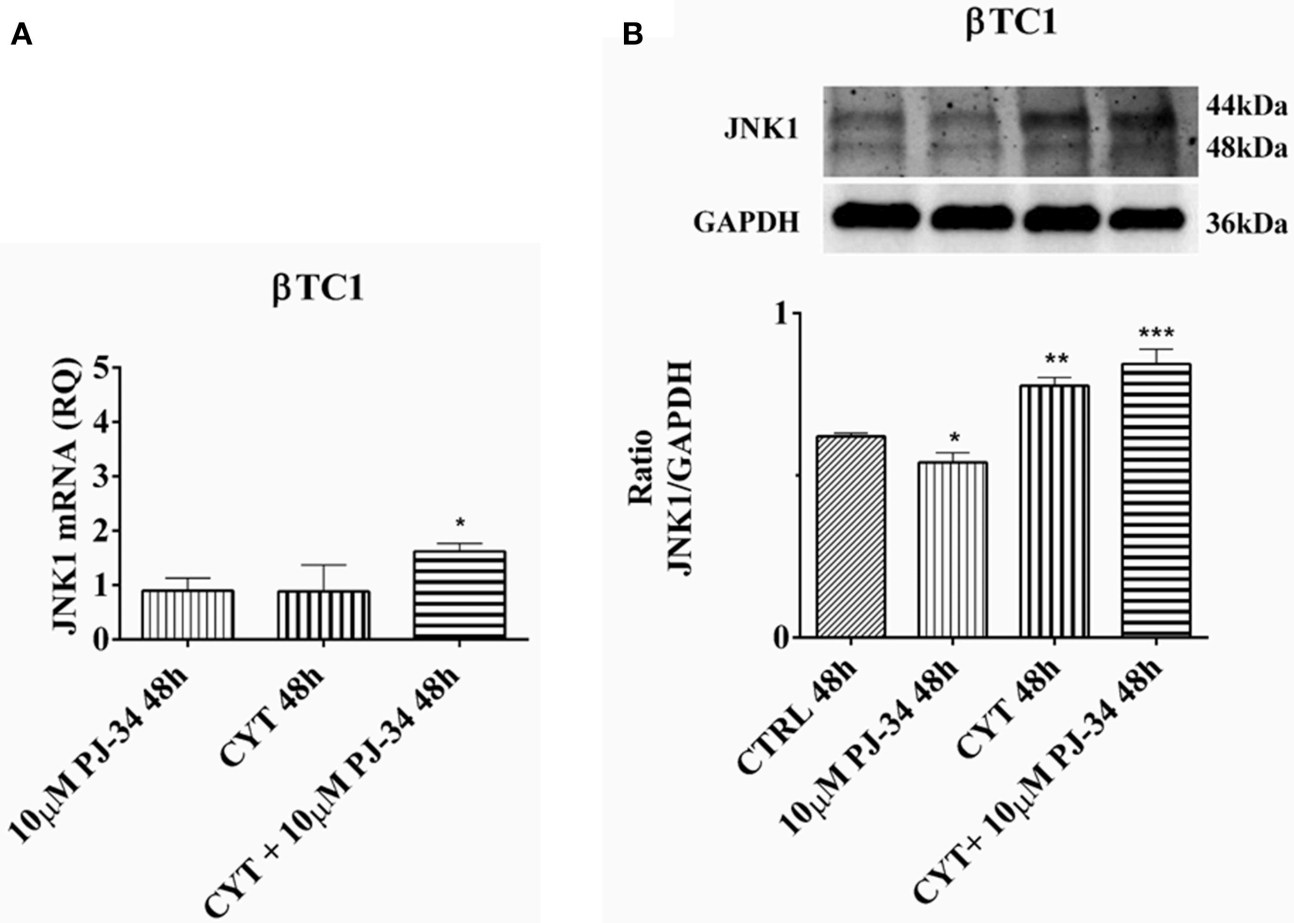
Effect of the PARP inhibitor PJ-34 on JNK1 mRNA and protein expression in βTC1 cells, grown for 48 h in the presence or absence of cytokines. Real-time PCR and total cell lysate immunoblottings were performed as described in the Materials and Methods section. βTC1 cells were grown: in normal culture medium (control: CTRL); in the presence of 10 μM PJ-34; in culture medium containing cytokine cocktail (CYT: TNF-α 25 U/ml; IFN-γ 25 U/ml; IL-1β 0.1 U/ml); in culture medium with the addition of both cytokine cocktail and 10 μM PJ-34 (CYT + 10 μM PJ-34), for 48 h. **A**. Relative quantity (RQ) level of JNK1 mRNA, at 48 h, in the experimental conditions mentioned above. Relative quantification is referred to untreated cells. **(B)** JNK1 protein was revealed with a rabbit polyclonal antibody (1:5000 dilution) as described in Materials and Methods section. The blots were controlled for equal loading by GAPDH, using a mouse monoclonal antibody (1:2000 dilution). Immunoreactive bands were visualized by chemiluminescence (ECL system). The values were obtained by the reading of blots through the Image J program. Statistical analysis was carried out by One-way Anova test, using control (CTRL) and cytokines (CYT) conditions as reference samples. The bars represent means ± SD of three independent experiments (S.D. = standard deviation). Asterisks represent a significant difference between the treated samples and CTRL (^***^p < 0.001; ^**^p < 0.01; ^*^p < 0.05).

In βTC1 cells, no differences in JNK1 mRNA expression levels were observed at 24 h (Figure S4A). However, at the same time point, cytokines was able to induce a significant increase of JNK1 protein (Figure S4B). Cytokines and PJ-34, in combination, down-regulated JNK1 protein levels (Figure S4B). At 48 h, it is possible to observe a significant increment of JNK1 protein in the presence of cytokines alone and a significant increase of both mRNA and protein levels in the presence of the combination of cytokines with 10 μM PJ-34, compared with control (Figures 8A,B).

### Effect of the PARP Inhibitor PJ-34 on JNK2 mRNA and Protein Expression in αTC1.6 Cells, Grown for 24 and 48 h in the Presence or Absence of Cytokines

**Figure 9:**
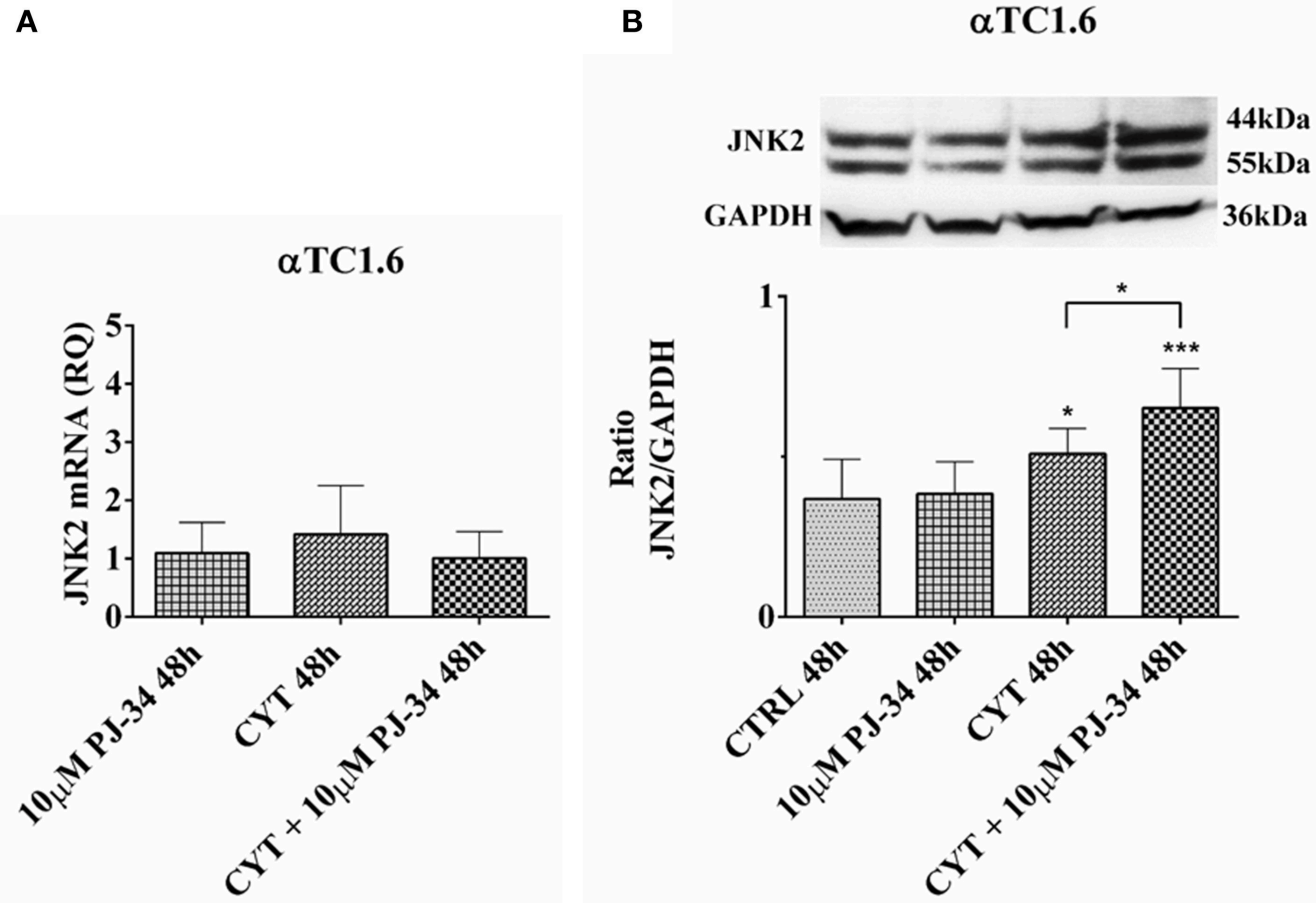
Effect of the PARP inhibitor PJ-34 on JNK2 mRNA and protein expression in αTC1.6 cells, grown for 48 h in the presence or absence of cytokines. Real-time PCR and total cell lysate immunoblottings were performed as described in the Materials and Methods section. αTC1.6 cells were grown: in normal culture medium (control: CTRL); in the presence of 10 μM PJ-34; in culture medium containing cytokine cocktail (CYT: TNF-α 25 U/ml; IFN-γ 25 U/ml and IL-1β 0.1 U/ml); in culture medium with the addition of both cytokine cocktail and 10 μM PJ-34 (CYT + 10 μM PJ-34), for 48 h. **(A)** Relative quantity (RQ) level of JNK2 mRNA, at 48 h, in the experimental conditions mentioned above. Relative quantification is referred to untreated cells. **(B)** JNK2 protein was revealed with a rabbit polyclonal antibody (1:4000 dilution) as described in Materials and Methods section. The blots were controlled for equal loading by GAPDH, using a mouse monoclonal antibody (1:2000 dilution). Immunoreactive bands were visualized by chemiluminescence (ECL system).The values were obtained by the reading of blots through the Image J program. Statistical analysis was carried out by One-way Anova test, using control (CTRL) and cytokines (CYT) conditions as reference samples. The bars represent means ± SD of three independent experiments (S.D. = standard deviation). Asterisks represent a significant difference between the treated samples and CTRL. The significance between CYT +10 μM PJ-34 and CYT is indicated by the asterisks upon the sticks (^***^p < 0.001; ^*^p < 0.05).

No notable effect was found on JNK2 mRNA expression levels in all experimental conditions, at both 24 h (Figure S5A) and 48 h (Figure 9A). Conversely, JNK2 protein expression levels were significantly reduced when αTC1.6 were grown in the presence of both cytokines and PJ-34, compared with control and cytokines alone, for 24 h (Figure S5B). At 48 h, a significant increase of JNK2 expression was detected in the presence of cytokines compared with the control and in presence of the combination of cytokines with 10 μM PJ-34 compared with both control and cytokines alone (Figure 9B).

### Effect of the PARP Inhibitor PJ-34 on JNK2 mRNA and Protein Expression in βTC1 Cells, Grown for 24 and 48 h in the Presence or Absence of Cytokines

**Figure 10:**
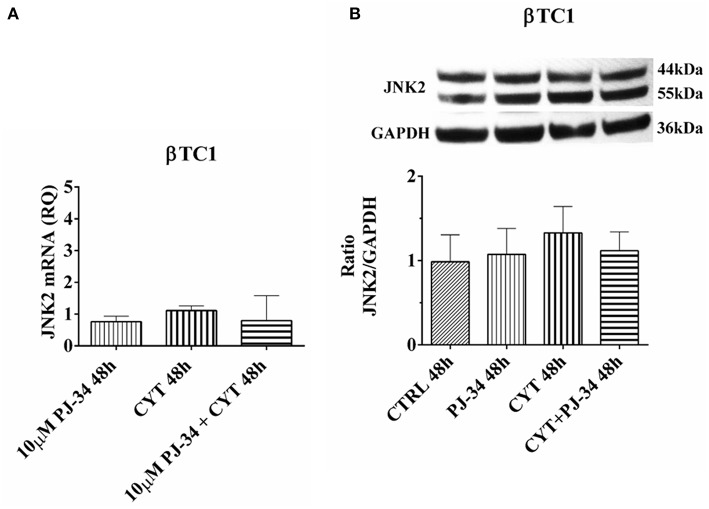
Effect of the PARP inhibitor PJ-34 on JNK-2 mRNA and protein expression in βTC1 cells, grown for 48 h in the presence or absence of cytokines. Real-time PCR and total cell lysate immunoblottings were performed as described in the Materials and Methods section. βTC1 cells were grown: in normal culture medium (control: CTRL); in the presence of 10 μM PJ-34; in culture medium containing cytokine cocktail (CYT: TNF-α 25 U/ml; IFN-γ 25 U/ml and IL-1β 0.1 U/ml); in culture medium with the addition of both cytokine cocktail and 10 μM PJ-34 (CYT + 10 μM PJ-34), for 48 h. **(A)** Relative quantity (RQ) level of JNK2 mRNA, at 48 h, in the experimental conditions mentioned above. Relative quantification is referred to untreated cells. **(B)** JNK2 protein was revealed with a rabbit polyclonal antibody (1:4000 dilution) as described in Materials and Methods section. The blots were controlled for equal loading by GAPDH, using a mouse monoclonal antibody (1:2000 dilution). Immunoreactive bands were visualized by chemiluminescence (ECL system). The values were obtained by the reading of blots through the Image J program. Statistical analysis was carried out by One-way Anova test, using control (CTRL) and cytokines (CYT) conditions as reference samples. The bars represent means ± SD of three independent experiments (S.D. = standard deviation).

In βTC1 cells, JNK2 mRNA and protein expression levels did not show any significant variation in our experimental conditions, at both 24 h (Figures S6A,B) and 48 h (Figures 10A,B).

### Effect of the PARP Inhibitor PJ-34 on p53 mRNA and p53 Phosphorylated Protein Levels in αTC1.6, Grown for 24 and 48 h in the Presence or Absence of Cytokines

To verify the activation of the apoptotic cascade, in our experimental conditions, we analyzed the mRNA expression and the phosphorylation levels of p53 in both cell lines.

**Figure 11:**
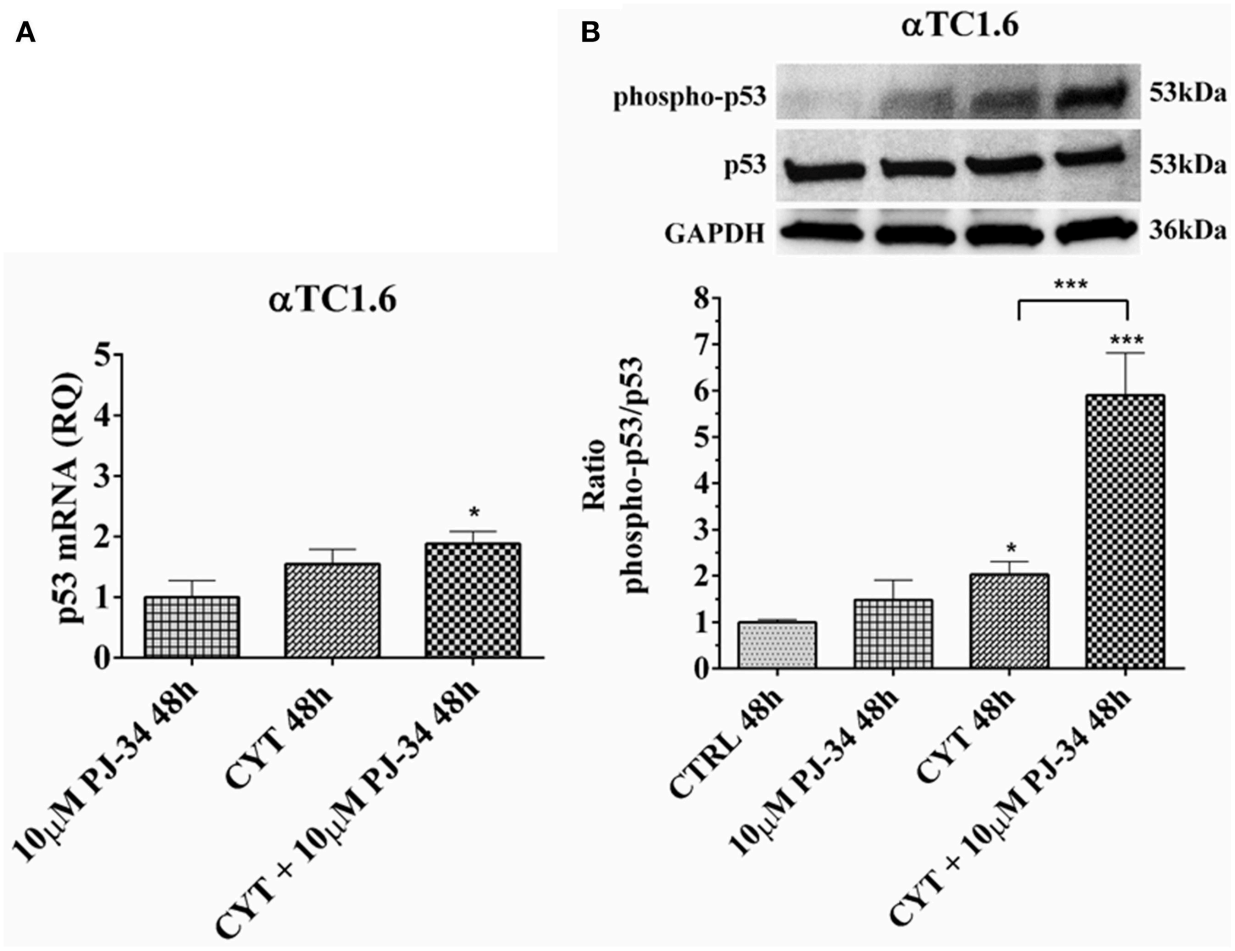
Effect of the PARP inhibitor PJ-34 on p53 mRNA expression and p53 phosphorylation level in αTC1.6 cells, grown for 48 h in the presence or absence of cytokines. Real-time PCR and total cell lysate immunoblottings were performed as described in the Materials and Methods section. αTC1.6 cells were grown: in normal culture medium (control: CTRL); in the presence of 10 μM PJ-34; in culture medium containing cytokine cocktail (CYT: TNF-α 25 U/ml; IFN-γ 25 U/ml and IL-1β 0.1 U/ml); in culture medium with the addition of both cytokine cocktail and 10 μM PJ-34 (CYT + 10 μM PJ-34), for 48 h. **(A)** Relative quantity (RQ) level of p53 mRNA, at 48 h, in the experimental conditions mentioned above. Relative quantification is referred to untreated cells. **(B)** The phosphorylation level of p53 protein was revealed with a rabbit polyclonal antibody (1:1000 dilution) as described in Materials and Methods section. The phosphorylated form of p53 was normalized with the total protein, using a mouse monoclonal antibody (1:1000 dilution). The blots were controlled for equal loading by GAPDH, using a mouse monoclonal antibody (1:2000 dilution). Immunoreactive bands were visualized by chemiluminescence (ECL system).The values were obtained by the reading of blots through the Image J program. Statistical analysis was carried out by One-way Anova test, using control (CTRL) and cytokines (CYT) conditions as reference samples. The bars represent means ± SD of three independent experiments (S.D. = standard deviation). Asterisks represent a significant difference between the condition and CTRL. The significance between CYT +10 μM PJ-34 and CYT is indicated by the asterisks upon the sticks (^***^p < 0.001; ^*^p < 0.05).

In α-cells, no significant variation of both p53 mRNA and protein levels were noted at 24 h, in the conditions tested (Figures S7A,B). At 48 h, cytokine treatment caused a significant increment of the p53 phosphorylated form vs. control (Figure 11B). At the same time point, the presence of both cytokines and PJ-34 induced a significant increase of mRNA compared with the control and the phosphorylated protein against control and cytokines alone (Figures 11A,B).

### Effect of the PARP Inhibitor PJ-34 on p53 mRNA and p53 Phosphorylated Protein Levels in βTC1 Cells, Grown for 24 and 48 h in the Presence or Absence of Cytokines

**Figure 12:**
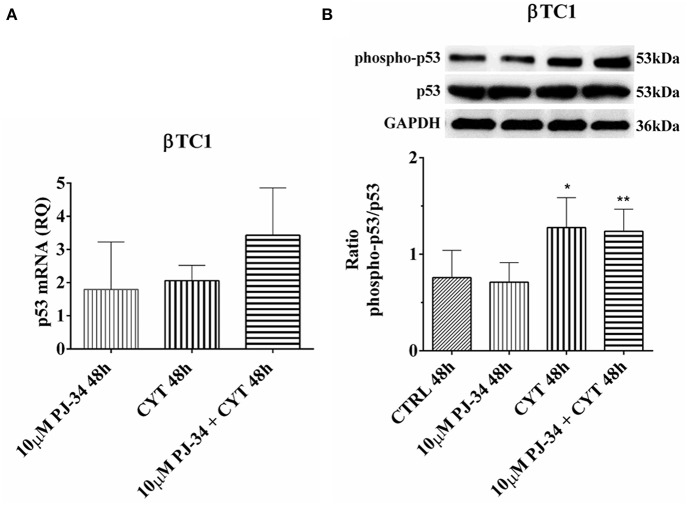
Effect of the PARP inhibitor PJ-34 on p53 mRNA expression and p53 phosphorylation level in βTC1 cells, grown for 48 h in the presence or absence of cytokines. Real-time PCR and total cell lysate immunoblottings were performed as described in the Materials and Methods section. βTC1 cells were grown: in normal culture medium (control: CTRL); in the presence of 10 μM PJ-34; in culture medium containing cytokine cocktail (CYT: TNF-α 25 U/ml; IFN-γ 25 U/ml and IL-1β 0.1 U/ml); in culture medium with the addition of both cytokine cocktail and 10 μM PJ-34 (CYT + 10 μM PJ-34), for 48 h. **(A)** Relative quantity (RQ) level of p53 mRNA, at 48 h, in the experimental conditions mentioned above. Relative quantification is referred to untreated cells. **(B)** The phosphorylation level of p53 protein was revealed with a rabbit polyclonal antibody (1:1000 dilution) as described in Materials and Methods section. The phosphorylated form of p53 was normalized with the total protein, using a mouse monoclonal antibody against total p53 (1:1000 dilution). The blots were controlled for equal loading by GAPDH, using a mouse monoclonal antibody (1:2000 dilution). Immunoreactive bands were visualized by chemiluminescence (ECL system). The values were obtained by the reading of blots through the Image J program. Statistical analysis was carried out by One-way Anova test, using control (CTRL) and cytokines (CYT) conditions as reference samples. The bars represent means ± SD of three independent experiments (S.D. = standard deviation). Asterisks represent a significant difference between the condition and CTRL (^**^p < 0.01; ^*^p < 0.05).

In βTC1 cells, at 24 h, no significant variation was detected in p53 mRNA expression and in its phosphorylation level (Figures S8A,B). At 48, the inflammatory state did not induce a significant variation in mRNA expression (Figure 12A). However, it is evident that the p53 phosphorylation level in the presence of cytokines and cytokines + 10 μM PJ-34 doubled the value of control (Figure 12B). This could indicate that, at this time point, cytokine stimulation is able to trigger the apoptotic death of βTC1 cells, mediated by the p53 protein, independently of the presence of PJ-34.

## Discussion

**Figure 13:**
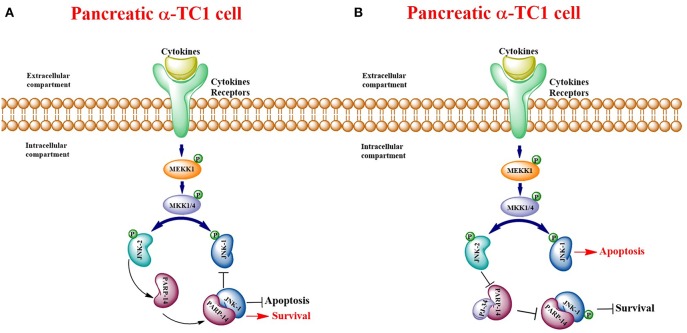
Schematic model of pancreatic αTC1.6 cells pathways. Binding of cytokines to their specific receptors activates the Mitogen-Activated Protein Kinase (MAPK) signaling cascade: this culminates in the activation of the Stress-Activated Protein Kinases (SAPK)/c Jun N-terminal Kinases (JNK). Therefore, in presence of inflammatory stimuli, JNK-2 activates PARP-14, which binds to and counteracts the pro-apoptotic protein JNK-1, promoting cell survival **(A)**. PARP-14, bound to the inhibitor PJ-34, is not able to interact with other molecules and JNK-1 can trigger apoptotic death **(B)**.

PARP family members are involved in a large variety of physiological and pathological events (5, 31, 32). Until now, most studies have focused on PARP-1, initially as an enzyme involved only in DNA repair, but subsequently also in apoptotic and necrotic cell death and in cancer (4, 33, 34). Nevertheless, what is biologically interesting about these proteins is that they can play a key role also in cell proliferation and survival (35). In fact, in a previous paper, we published data on the involvement of PARP-1 in a DNA independent way within the Extracellular-Regulated-Kinase (ERK) signaling cascade, which regulates cell proliferation and migration of rat brain microvascular endothelial cells. Furthermore, we showed the ability of the PARP inhibitor PJ-34 to regulate PARP-1 mRNA and protein expression. Indeed, by binding the catalytic site of the enzyme, PJ-34 was able to affect both the enzymatic functions and PARP binding to target proteins (1, 36). Many published studies report that PARP inhibitors promote cancer cell death, as they block the process of repairing damaged DNA (27, 34, 37–41). However, these studies mainly concern PARP-1 (1–3, 42, 43). Despite this, other members of the PARP superfamily could also play a decisive role in regulating various cellular processes including inflammation and immunity (44). In this study, the authors demonstrated that PARP-14 represents an important co-factor of signal transduction and activation of transcription 6 (STAT-6), which is involved in B cell and T cell responses to interleukin-4 (IL-4), mainly in the differentiation of T helper type 2 (Th2) cells. Furthermore, in PARP-14 deficiency mice or those subjected to pharmacological block of PARP-14 activity, the development of T follicular helper (Tfh) cells and the differentiation of Th17 cells are compromised in both *in vitro* and *in vivo* models (44). A further study reports that PARP-14 is involved in the survival pathway in multiple myeloma (MM) (16). Very interesting data in this paper showed that PARP-14 overexpression totally prevented myeloma cells from undergoing apoptosis, induced by JNK2 knockdown: this suggests that PARP-14 is essential in JNK2 dependent survival signaling. PARP-14 is able to bind and inhibit JNK1 that promotes apoptosis by phosphorylating several downstream transcription factors (21, 45). In our study, the expression analysis of murine PARP family members by qPCR allowed us to highlight an over-expression of many PARPs in both cell lines, under inflammatory state. Nevertheless, we focused on PARP-14 because: (1) its induction was significantly higher in αTC1.6 than in βTC1, after treatment with cytokines, (see Table 2); (2) recent literature data report its involvement in a survival pathway that can justify a critical role played by PARP-14 in α cell survival (16, 17). Through expression studies, carried out by qPCR, western blot and confocal analysis, we demonstrated that PARP-14 is activated after cytokine treatment in α and β cells. A possible link between PARP-14 and interleukin was described (15). In this paper, they demonstrated that IL-4 protection of B cells from apoptosis depends on PARP-14. In our model, treatment of the two cell types with cytokines caused cell death only of βTC1 cells. β cell loss is traditionally considered a major cause of type I diabetes onset. On the other hand, a concomitant role of glucagon secreting pancreatic α cells in the pathogenesis of type I diabetes has been proposed (46–48). As is well-documented, both α and β cells have a common origin, but the latter are more vulnerable to apoptosis under inflammatory conditions, which are common in type I diabetes (20). In this report, the authors suggested that JNK1 is a key mediator of IL-1β-induced apoptosis in a rat β-cell line and that it is able to modulate apoptosis through the transcription factor Myc. Another study demonstrated that the use of JNK inhibitor prevents human β cells from apoptosis, induced by glucose and leptins through the activation of JNK (49). Therefore, since PARP-14 is involved in a transduction pathway mediated by JNKs, promoting survival in multiple myeloma (16), we hypothesized the activation of this signaling pathway also in our αTC1.6 cell line, in an inflammatory experimental model. Indeed, the activation of this protective pathway could explain the resistance of α cells to apoptosis induced by cytokines. For this reason, since β cells are more vulnerable to an inflammatory environment and express lower levels of PARP-14 than α cells, we considered β cells a useful comparison system. Our results deepen our knowledge regarding the role played by JNKs and PARP-14 in pancreatic αTC1.6 and in βTC1 cells, in an inflammatory state. In particular, JNK1 expression levels showed a different behavior in αTC1.6 compared to βTC1 cells. In fact, in αTC1.6 cells, the inflammatory state did not determine any significant increase in JNK1 expression, proving that cytokines were not able to trigger the apoptotic cascade mediated by this protein. Conversely, in the same conditions, βTC1 cells up-regulate JNK1 expression and p53 phosphorylation mediated by JNK1, thus activating apoptosis. Furthermore, at 48 h, under inflammatory conditions and in the presence of PJ-34, α cells overexpressed JNK1, responsible for p53 phosphorylation. These data show that, when PARP-14 is inhibited, the role played by JNK1 in inducing apoptosis prevails. In addition, the ability of PJ-34 to make αTC1.6 cells susceptible to apoptotic death induction by cytokines was confirmed by flow cytometry. In fact, the reduction of vital cells and the concomitant increase of early apoptotic cell populations were found only in the presence of PJ-34. On the contrary, in β cells, the reduction of early apoptotic cell rate was followed by an increase of the late apoptotic cell rate, indicating that β cells go to an irreversible apoptotic cell death. These results are consistent with the expression levels of JNK1. Moreover, the addition of PJ-34 reduced JNK1 expression and p53 phosphorylation, only at 24 h. However, since the reduction of these proteins was not followed by an increase of β cell survival (see flow cytometry results), we could hypothesize that, unlike α cells, β cell death might not be dependent only on the JNK1-p53 pathway. On the other hand, further studies are needed to investigate the role played by PARP inhibitor PJ-34 and these two pro-apoptotic proteins in the βTC1 cell line. Further proof of the protective role of PARP-14 as well as the absence of susceptibility of αTC1.6 cells to apoptotic death, induced by inflammation, was provided by the caspase-3 activity assay. As was reported, PARP-14 promotes survival by inhibiting caspase activity (17). It was demonstrated that IL-4 is less efficient in reducing caspase activity in PARP-14 KO B cells. Therefore, in our model, cytokine stimulation, in α cells, was not able to induce any variation of caspase-3 activity at both time points, demonstrating, once again, the resistance of this cell line to inflammatory insults. In fact, the increase of caspase-3 catalytic ability, in α cell, occurred only at 48 h, when PARP-14 is blocked by PJ-34. In this case, the caspase-3 catalytic activity variation is due to PARP-14 inhibition by PJ-34 and not to cytokine stimulation. Instead, β cells appear to be susceptible to the action of cytokines, as demonstrated by the increase of caspase-3 activity levels, which were maintained after the addition of PJ-34. It seems clear that, in these cells, PJ-34 did not affect caspase-3 activity, proving that it acts in a different way than in αTC1.6 cells. Finally, through the expression patterns of JNK2 and PARP-14, we demonstrated that the resistance of α cells to an inflammatory environment is due to the activation of the JNK2/PARP-14 survival pathway. The mRNA trend and confocal analysis showed that αTC1.6 cells overexpressed PARP-14 only when they were stimulated by cytokines, mainly at 48 h. However, at this time point, the addition of the PARP inhibitor PJ-34 was able to counteract the increase of PARP-14 expression, induced by cytokines. At 48 h, the inflammatory state also caused a significant increment of JNK2 expression that was higher in the presence of PJ-34. Briefly, when PARP-14 is blocked, JNK2 is further up-regulated, favoring the beginning of the survival signaling cascade. Instead, the down-regulation of JNK2 mediated by PJ-34 at 24 h could indicate that, at this time point, the inflammatory stimulation is not adequate to trigger a survival pathway, as was demonstrated by flow cytometry. In β cells, the trend of PARP-14 and JNK2 clearly confirms that this signaling cascade was not engaged. All these data, taken together, demonstrate that PARP-14 is definitely involved in the resistance of pancreatic α cells to inflammatory insults, through the transduction pathway mediated by JNK proteins promoting survival or apoptosis. In this regard, we hypothesized that the interaction between PARP-14 and its inhibitor could impede the protein binding JNK1, which should be free to trigger the apoptosis cascade (see schematic model in Figure 13). Certainly, further work will be required to elucidate other mechanisms involved in this complex signal transduction pathway.

## Conclusion

This study must be seen as a first piece of a puzzle where PARP-14, JNKs and PJ-34 play key roles in the pancreatic microenvironment and provide starting points from which to explore further.

## Author Contributions

VS-P conceived the project and designed the experiments together with MP and CD. MR and FD performed molecular experiments as RT-PCR and western analysis. CS and MS performed confocal analysis. FD, NM, and VB performed cytofluorimetric analysis. FD and MC were in charge of cell culture and treatment. FD performed computational and statistical data analysis, together with AT-S. VS-P and FD wrote the paper. All authors contributed to the critical revision of the data, read and approved the final manuscript.

### Conflict of Interest Statement

The authors declare that the research was conducted in the absence of any commercial or financial relationships that could be construed as a potential conflict of interest.
