# Supplementary material for: PARP-14 Promotes Survival of Mammalian α but Not β Pancreatic Cells Following Cytokine Treatment
Source: Front Endocrinol (Lausanne). 2019 May 3;10:271. doi: 10.3389/fendo.2019.00271 (PMC6509146; doi:10.3389/fendo.2019.00271)
Supplement: Supplementary file 1 [file Data_Sheet_1.docx]

**Table S1. Fold change values of fifteen PARP family members in murine pancreatic αTC1.6 and βTC1 cells after 24h of cytokine treatment.** Fold change values (Avg FC) of each PARP are reported comparing PARP ΔCt values of the cells treated with the cytokine (CYT) cocktail (TNF-α 25 U/ml; IFN-γ 25 U/ml and IL-1β 0.1 U/ml) and PARP ΔCt values of steady state cells (Control: CTRL), at 24h. qPCR experiments were carried out in triplicate (n = 3). Statistical significance was determined with Student’s t-test. Std Dev and p-value columns indicate the standard deviation values and the significance of every single PARP, respectively.

| **PARP family member** | **Avg FC αTC1.6 CYT 24h** | **Std Dev** | **p-value** | **Avg FC βTC1 CYT 24h** | **Std Dev** | **p-value** |
| --- | --- | --- | --- | --- | --- | --- |
| Parp1 | 0.79 | ± 0.31 | 0.88932 | 1.37 | ± 0.43 | 0.126506 |
| Parp2 | 0.66 | ± 0.24 | 0.18911 | 1.74 | ± 0.73 | 0.208393 |
| Parp3 | 2.65 | ± 0.86 | 0.79956 | 8.2 | ± 1.67 | 0.038244 |
| Parp4 | 0.55 | ± 0.32 | 0.83722 | 1.57 | ± 0.08 | 0.030728 |
| Tnks | 0.45 | ± 0.23 | 0.16155 | 1.53 | ± 0.57 | 0.186523 |
| Tnks2 | 0.55 | ± 0.23 | 0.41073 | 1.1 | ± 0.06 | 0.172074 |
| Parp6 | 0.79 | ± 0.21 | 0.09718 | 1.64 | ± 0.43 | 0.527909 |
| Parp7 | 0.64 | ± 0.14 | 0.21174 | 1.06 | ± 0.23 | 0.607469 |
| Parp8 | 0.52 | ± 0.23 | 0.08341 | 1.72 | ± 0.30 | 0.532938 |
| Parp9 | 19.94 | ± 0.65 | 0.00000014 | 21.41 | ± 0.27 | 0.000000012 |
| Parp10 | 4.86 | ± 0.68 | 0.01318 | 5.95 | ± 2.21 | 0.031146 |
| Parp11 | 5.77 | ± 0.26 | 0.00201 | 5.53 | ± 0.14 | 0.000115 |
| Parp12 | 2.79 | ± 0.65 | 0.00465 | 4.28 | ± 1.17 | 0.029558 |
| Parp14 | 2356.68 | ± 6.35 | 0.00017 | 129.99 | ± 0.98 | 0.00000025 |
| Parp16 | 0.91 | ± 0.31 | 0.93425 | 1.68 | ± 0.33 | 0.01482 |





**Figure S1. PARP-14 mRNA expression in murine pancreatic αTC1.6 and βTC1 cells following 24h of cytokine treatment.** Pancreatic αTC1.6 and βTC1 cells were grown in normal medium (Control: CTRL) or in the presence of cytokine cocktail (CYT: TNF-α 25 U/ml; IFN-γ 25 U/ml and IL-1β 0.1 U/ml), for 24h. Box and whisker plots represent PARP-14 mRNA expression levels in αTC1.6 and βTC1 cells exposed to inflammatory stimuli compared to their relative control. Y-axis represents the distribution of -1*ΔCt values for PARP-14 mRNA. The qPCR experiments were carried out in triplicate (n = 3). Statistical significance was determined with Student’s t-test, comparing the control ΔCt values (CTRL) to those of the cytokines (CYT). Asterisks represent a significant difference between the CYT and CTRL (*******p<0.001).

**
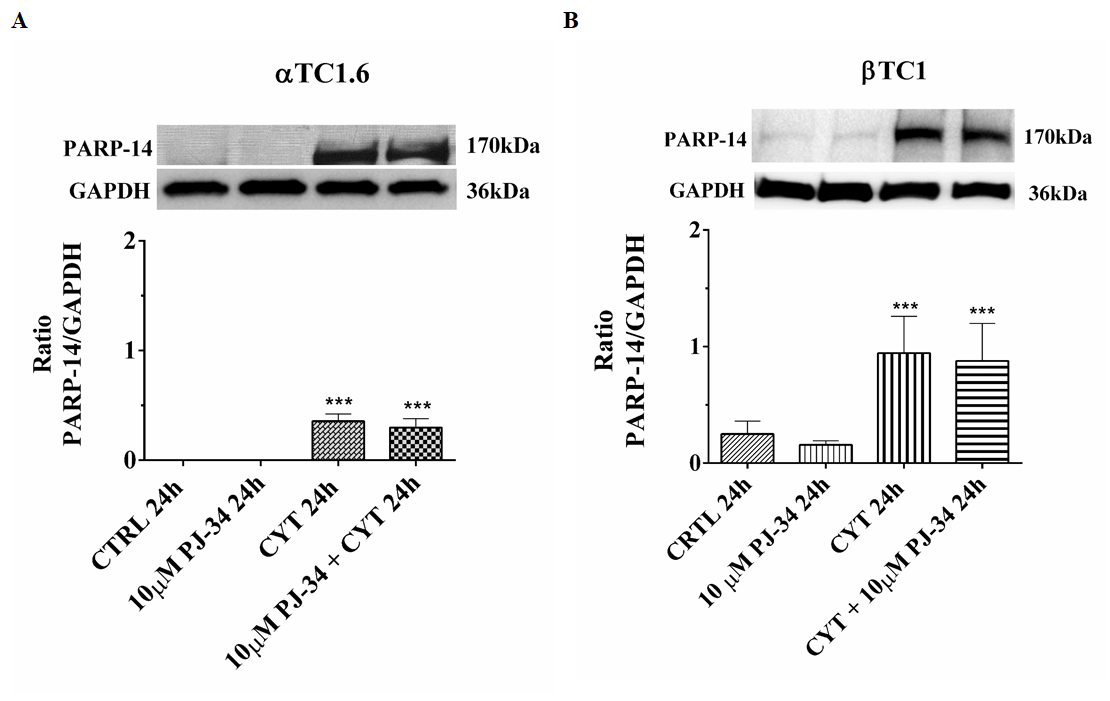
**

**Figure S2.** **Effect of the PARP inhibitor PJ-34 on PARP-14 expression in αTC1.6 and βTC1 cells, grown for 24h in the presence or absence of cytokines.** αTC1.6 (Figure S2A) and βTC1 (Figure S2B) cells were grown in normal culture medium: control (CTRL); in the presence of 10µM PJ-34; in culture medium containing cytokine cocktail (CYT: TNF-α 25 U/ml; IFN-γ 25 U/ml and IL-1β 0.1 U/ml); in culture medium with the addition of both cytokine cocktail and 10µM PJ-34 (CYT + 10µM PJ-34), for 24h. Expressed protein was revealed with a mouse monoclonal antibody against PARP-14 (1:500 dilution) as described in Materials and Methods section. The blots were controlled for equal loading by GAPDH, using a mouse monoclonal antibody (1:2000 dilution). Immunoreactive bands were visualized by chemiluminescence (ECL system).The values were obtained by the reading of blots using the Image J program. Statistical analysis was made using One-way Anova test, using control (CTRL) and cytokines (CYT) conditions as reference samples. The bars represent means ± SD of three independent experiments (S.D. = standard deviation). Asterisks represent a significant difference between the treated samples and CTRL. The significance between CYT +10µM PJ-34 and CYT is indicated by the asterisks upon the sticks (*******p<0.001).


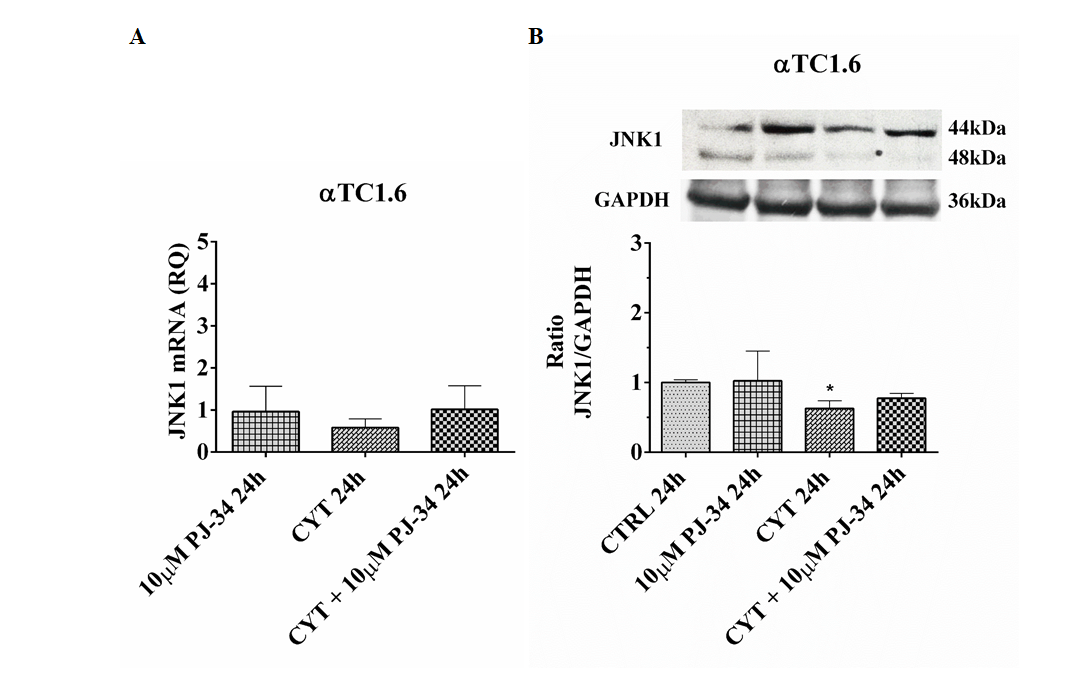


**Figure S3.** **Effect of the PARP inhibitor PJ-34 on JNK1 mRNA and protein expression in αTC1.6 cells, grown for 24h in the presence or absence of cytokines.** Real-time PCR and total cell lysate immunoblottings were performed as described in the Materials and Methods section. αTC1.6 cells were grown: in normal culture medium (control: CTRL); in the presence of 10µM PJ-34; in culture medium containing cytokine cocktail (CYT: TNF-α 25 U/ml; IFN-γ 25 U/ml and IL-1β 0.1 U/ml); in culture medium with the addition of both cytokine cocktail and 10µM PJ-34 (CYT + 10µM PJ-34), for 24h. **A.** Relative quantity (RQ) level of JNK1 mRNA, at 24h, in the experimental conditions mentioned above. Relative quantification is referred to untreated cells. **B.** JNK1 protein was revealed with a rabbit polyclonal antibody (1:5000 dilution) as described in Materials and Methods section. The blots were controlled for equal loading by GAPDH, using a mouse monoclonal antibody (1:2000 dilution). Immunoreactive bands were visualized by chemiluminescence (ECL system). The values were obtained by the reading of blots through the Image J program. Statistical analysis was carried out by One-way Anova test, using control (CTRL) and cytokines (CYT) conditions as reference samples. The bars represent means ± SD of three independent experiments (S.D. = standard deviation). Asterisks represent a significant difference between the treated samples and CTRL (*****p< 0.05).

**
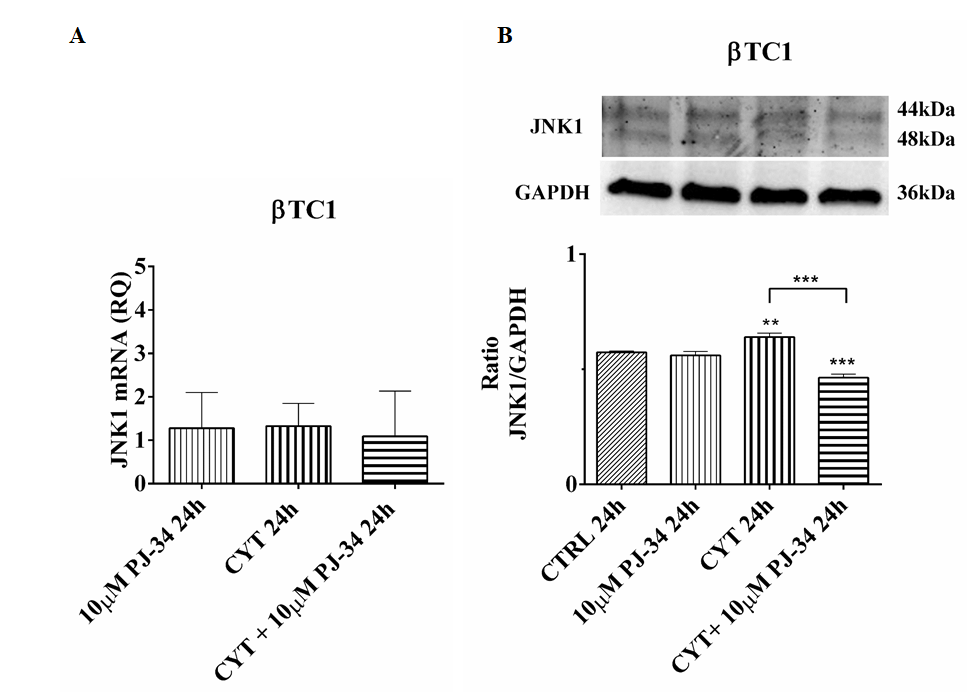
**

**Figure S4.** **Effect of the PARP inhibitor PJ-34 on JNK1 mRNA and protein expression in βTC1 cells, grown for 24h in the presence or absence of cytokines.** Real-time PCR and total cell lysate immunoblottings were performed as described in the Materials and Methods section. βTC1 cells were grown: in normal culture medium (control: CTRL); in the presence of 10µM PJ-34; in culture medium containing cytokine cocktail (CYT: TNF-α 25 U/ml; IFN-γ 25 U/ml; IL-1β 0.1 U/ml); in culture medium with the addition of both cytokine cocktail and 10µM PJ-34 (CYT + 10µM PJ-34), for 24h. **A.** Relative quantity (RQ) level of JNK1 mRNA, at 24h, in the experimental conditions mentioned above. Relative quantification is referred to untreated cells. **B.** JNK1 protein was revealed with a rabbit polyclonal antibody (1:5000 dilution) as described in Materials and Methods section. The blots were controlled for equal loading by GAPDH, using a mouse monoclonal antibody (1:2000 dilution). Immunoreactive bands were visualized by chemiluminescence (ECL system). The values were obtained by the reading of blots through the Image J program. Statistical analysis was carried out by One-way Anova test, using control (CTRL) and cytokines (CYT) conditions as reference samples. The bars represent means ± SD of three independent experiments (S.D. = standard deviation). Asterisks represent a significant difference between the treated samples and CTRL. The significance between CYT +10µM PJ-34 and CYT is indicated by the asterisks upon the sticks (*******p<0.001; ******p< 0.01).


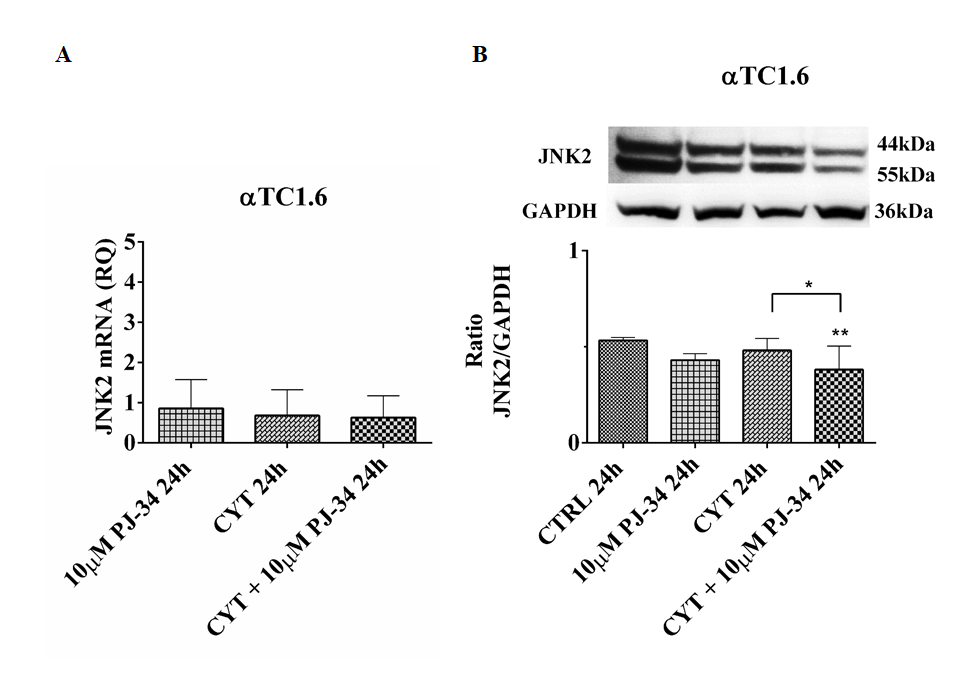


**Figure S5.** **Effect of the PARP inhibitor PJ-34 on JNK2 mRNA and protein expression in αTC1.6 cells, grown for 24h in the presence or absence of cytokines.** Real-time PCR and total cell lysate immunoblottings were performed as described in the Materials and Methods section. αTC1.6 cells were grown: in normal culture medium (control: CTRL); in the presence of 10µM PJ-34; in culture medium containing cytokine cocktail (CYT: TNF-α 25 U/ml; IFN-γ 25 U/ml and IL-1β 0.1 U/ml); in culture medium with the addition of both cytokine cocktail and 10µM PJ-34 (CYT + 10µM PJ-34), for 24h. **A.** Relative quantity (RQ) level of JNK2 mRNA, at 24h, in the experimental conditions mentioned above. Relative quantification is referred to untreated cells. **B.** JNK2 protein was revealed with a rabbit polyclonal antibody (1:4000 dilution) as described in Materials and Methods section. The blots were controlled for equal loading by GAPDH, using a mouse monoclonal antibody (1:2000 dilution). Immunoreactive bands were visualized by chemiluminescence (ECL system).The values were obtained by the reading of blots through the Image J program. Statistical analysis was carried out by One-way Anova test, using control (CTRL) and cytokines (CYT) conditions as reference samples. The bars represent means ± SD of three independent experiments (S.D. = standard deviation). Asterisks represent a significant difference between the treated samples and CTRL. The significance between CYT +10µM PJ-34 and CYT is indicated by the asterisks upon the sticks (******p<0.01; *****p< 0.05).


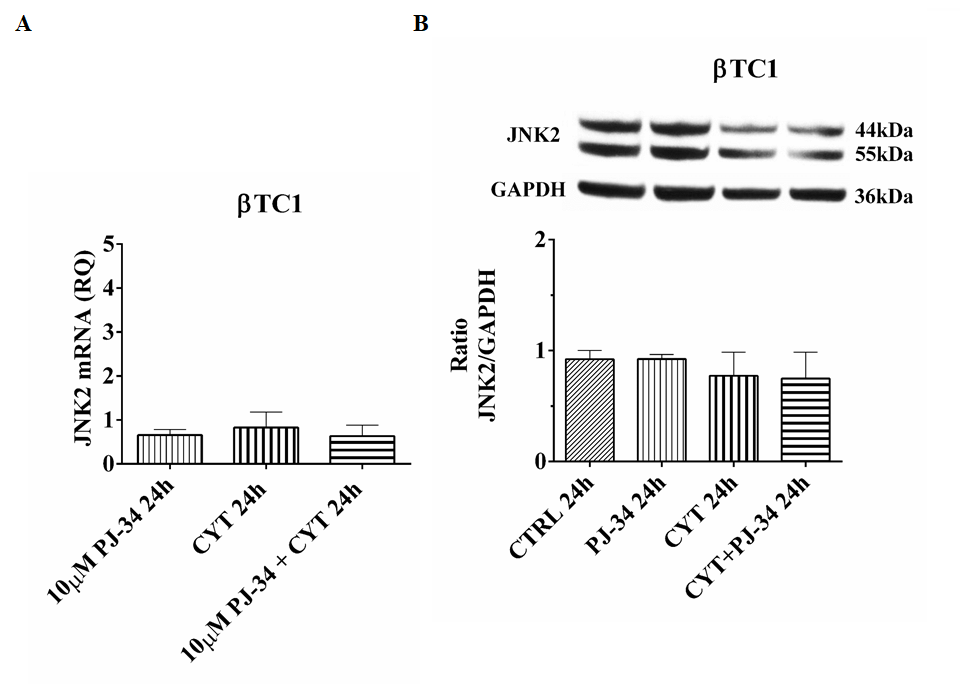


**Figure S6. Effect of the PARP inhibitor PJ-34 on JNK-2 mRNA and protein expression in βTC1 cells, grown for 24h in the presence or absence of cytokines.** Real-time PCR and total cell lysate immunoblottings were performed as described in the Materials and Methods section. βTC1 cells were grown: in normal culture medium (control: CTRL); in the presence of 10µM PJ-34; in culture medium containing cytokine cocktail (CYT: TNF-α 25 U/ml; IFN-γ 25 U/ml and IL-1β 0.1 U/ml); in culture medium with the addition of both cytokine cocktail and 10µM PJ-34 (CYT + 10µM PJ-34), for 24h. **A.** Relative quantity (RQ) level of JNK2 mRNA, at 24h, in the experimental conditions mentioned above**.** Relative quantification is referred to untreated cells. **B.** JNK2 protein was revealed with a rabbit polyclonal antibody (1:4000 dilution) as described in Materials and Methods section. The blots were controlled for equal loading by GAPDH, using a mouse monoclonal antibody (1:2000 dilution). Immunoreactive bands were visualized by chemiluminescence (ECL system). The values were obtained by the reading of blots through the Image J program. Statistical analysis was carried out by One-way Anova test, using control (CTRL) and cytokines (CYT) conditions as reference samples. The bars represent means ± SD of three independent experiments (S.D. = standard deviation).


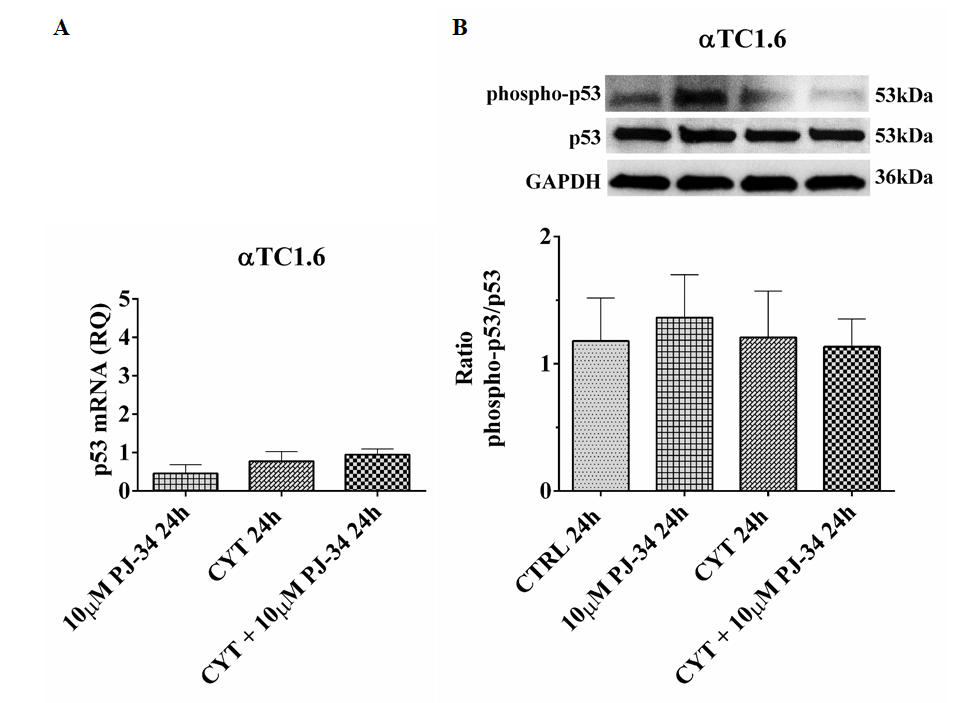


**Figure S7. Effect of the PARP inhibitor PJ-34 on p53 mRNA expression and p53 phosphorylation level in αTC1.6 cells,** **grown for 24h in the presence or absence of cytokines.** Real-time PCR and total cell lysate immunoblottings were performed as described in the Materials and Methods section. αTC1.6 cells were grown: in normal culture medium (control: CTRL); in the presence of 10µM PJ-34; in culture medium containing cytokine cocktail (CYT: TNF-α 25 U/ml; IFN-γ 25 U/ml and IL-1β 0.1 U/ml); in culture medium with the addition of both cytokine cocktail and 10µM PJ-34 (CYT + 10µM PJ-34), for 24h. **A.** Relative quantity (RQ) level of p53 mRNA, at 24h, in the experimental conditions mentioned above. Relative quantification is referred to untreated cells. **B.** The phosphorylation level of p53 protein was revealed with a rabbit polyclonal antibody (1:1000 dilution) as described in Materials and Methods section. The phosphorylated form of p53 was normalized with the total protein, using a mouse monoclonal antibody (1:1000 dilution). The blots were controlled for equal loading by GAPDH, using a mouse monoclonal antibody (1:2000 dilution). Immunoreactive bands were visualized by chemiluminescence (ECL system).The values were obtained by the reading of blots through the Image J program. Statistical analysis was carried out by One-way Anova test, using control (CTRL) and cytokines (CYT) conditions as reference samples. The bars represent means ± SD of three independent experiments (S.D. = standard deviation).


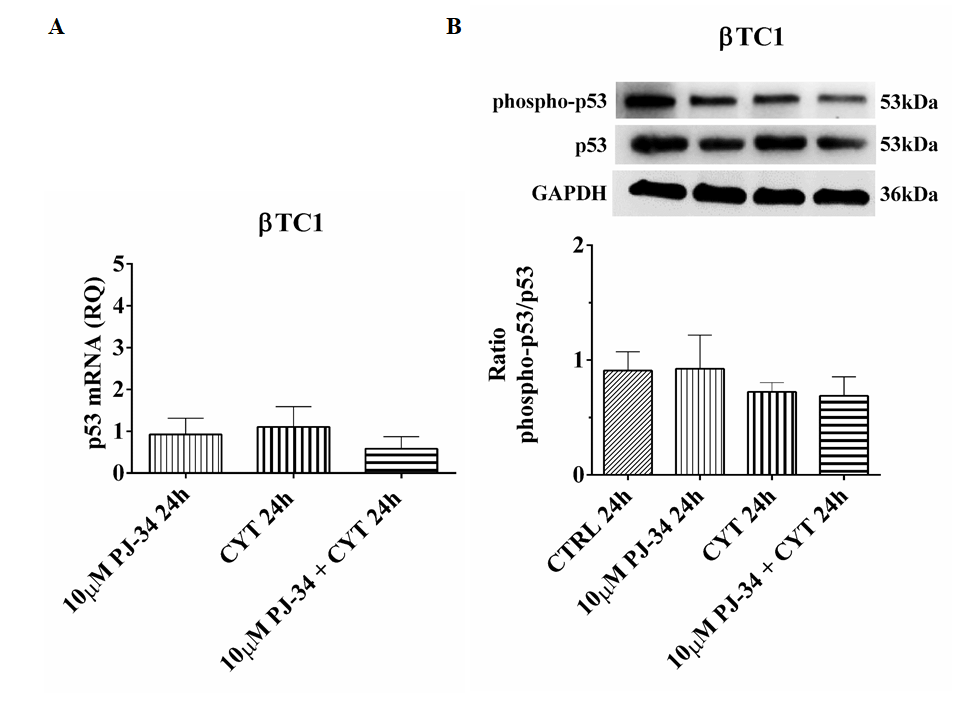


**Figure S8. Effect of the PARP inhibitor PJ-34 on p53 mRNA expression and p53 phosphorylation level in βTC1 cells, grown for 24h in the presence or absence of cytokines.** Real-time PCR and total cell lysate immunoblottings were performed as described in the Materials and Methods section. βTC1 cells were grown: in normal culture medium (control: CTRL); in the presence of 10µM PJ-34; in culture medium containing cytokine cocktail (CYT: TNF-α 25 U/ml; IFN-γ 25 U/ml and IL-1β 0.1 U/ml); in culture medium with the addition of both cytokine cocktail and 10µM PJ-34 (CYT + 10µM PJ-34), for 24h. **A.** Relative quantity (RQ) level of p53 mRNA, at 24h, in the experimental conditions mentioned above. Relative quantification is referred to untreated cells. **B.** The phosphorylation level of p53 protein was revealed with a rabbit polyclonal antibody (1:1000 dilution) as described in Materials and Methods section. The phosphorylated form of p53 was normalized with the total protein, using a mouse monoclonal antibody against total p53 (1:1000 dilution). The blots were controlled for equal loading by GAPDH, using a mouse monoclonal antibody (1:2000 dilution). Immunoreactive bands were visualized by chemiluminescence (ECL system). The values were obtained by the reading of blots through the Image J program. Statistical analysis was carried out by One-way Anova test, using control (CTRL) and cytokines (CYT) conditions as reference samples. The bars represent means ± SD of three independent experiments (S.D. = standard deviation).
